# Characterization of repeated DNA sequences in genomes of blue-flowered flax

**DOI:** 10.1186/s12862-019-1375-6

**Published:** 2019-02-26

**Authors:** Nadezhda L. Bolsheva, Nataliya V. Melnikova, Ilya V. Kirov, Alexey A. Dmitriev, George S. Krasnov, Аlexandra V. Amosova, Tatiana E. Samatadze, Olga Yu. Yurkevich, Svyatoslav A. Zoshchuk, Anna V. Kudryavtseva, Olga V. Muravenko

**Affiliations:** 1 Engelhardt Institute of Molecular Biology, Russian Academy of Sciences, Moscow, Russia; 2 Shemyakin-Ovchinnikov Institute of Bioorganic Chemistry, Russian Academy of Sciences, Moscow, Russia

**Keywords:** Genus *Linum*, Flax genome, High-throughput sequencing, Repeatome

## Abstract

**Background:**

Members of different sections of the genus *Linum* are characterized by wide variability in size, morphology and number of chromosomes in karyotypes. Since such variability is determined mainly by the amount and composition of repeated sequences, we conducted a comparative study of the repeatomes of species from four sections forming a clade of blue-flowered flax. Based on the results of high-throughput genome sequencing performed in this study as well as available WGS data, bioinformatic analyses of repeated sequences from 12 flax samples were carried out using a graph-based clustering method.

**Results:**

It was found that the genomes of closely related species, which have a similar karyotype structure, are also similar in the repeatome composition. In contrast, the repeatomes of karyologically distinct species differed significantly, and no similar tandem-organized repeats have been identified in their genomes. At the same time, many common mobile element families have been identified in genomes of all species, among them, Athila Ty3/gypsy LTR retrotransposon was the most abundant. The 30-chromosome members of the sect. *Linum* (including the cultivated species *L. usitatissimum*) differed significantly from other studied species by a great number of satellite DNA families as well as their relative content in genomes.

**Conclusions:**

The evolution of studied flax species was accompanied by waves of amplification of satellite DNAs and LTR retrotransposons. The observed inverse correlation between the total contents of dispersed repeats and satellite DNAs allowed to suggest a relationship between both classes of repeating sequences. Significant interspecific differences in satellite DNA sets indicated a high rate of evolution of this genomic fraction. The phylogenetic relationships between the investigated flax species, obtained by comparison of the repeatomes, agreed with the results of previous molecular phylogenetic studies.

**Electronic supplementary material:**

The online version of this article (10.1186/s12862-019-1375-6) contains supplementary material, which is available to authorized users.

## Background

Repeated sequences are important components of eukaryotic genomes, and often constitute a significant part of plant genomes [1]. They can be dispersed across the genome or arranged in large stretches of tandem repeat sequences (satellite DNA). Repeated sequences are predominantly located in functionally important regions of eukaryotic chromosomes (e.g., centromeres, telomeres and some chromosomal interstitial regions) being the main constituent of heterochromatic bands [2–4]. Dispersed repeat sequences often occupy a significant fraction of genomes being one of the major determinants of genome size differences in eukaryotes. They are mainly represented by different classes of transposable elements (TEs) capable of sometimes moving from one location in the genome to another. Since it has been shown that TE insertion can directly affect the gene function, it is suggested that activation of TEs movements in response to environmental stress, and the associated generation of new mutations may play some role in the process of adaptation of an organism to a changing environment [5–10]. Satellite sequences are the most rapidly evolving part of the eukaryotic genome. Functions of satellite sequences are still not clear enough but there is evidence of their involvement in the spatial chromosome organization, the mechanisms of chromosome pairing and segregation [11]. It was found that satellite DNA transcripts participate in formation and maintenance of heterochromatin structure [12]. It is suggested that the high rate of variability of centromeric satellite DNAs can contribute to speciation, creating interpopulation reproductive barriers [13, 14]. Even though the study of repetitive sequences is essential for understanding the principles of functional regulation and evolution of genomes, until recently information on the structure, organization and abundance of repetitive sequences in genomes was very fragmentary. This situation was mainly related to the technical difficulties of studying the repetitive elements in genomes [15]. The further development of NGS methods and new bioinformatic approaches has made it possible to change this situation radically. In particular, a set of software tools called “Repeat Explorer”, which allows identifying repetitive sequences using raw WGS reads [16, 17], was developed. Based on this approach, the repeatomes of many plant species have already been successfully investigated [18–27].

The genus *Linum* L. (*Linacea*) includes about 200 wild species. It’s assumed that the genus originated in Eurasia, and later it spreaded to Africa, Australia, North and South America. Recent molecular phylogenetic studies have shown that the genus *Linum* L. is not monophyletic but includes representatives of two sister clades: yellow-flowered and blue-flowered flax [28, 29]. Besides, each of these clades is subdivided into several groups of closely related species. So, yellow-flowered flax includes groups of species corresponding to the sect. *Cathartolinum* (Reichen b.) Griseb., *Syllinum* Griseb., *Linopsis* (Reichenb.) Engelmann and the genera *Cliococca* Bab., *Hesperolinon* (A. Gray) Small, *Radiola* Hill. and *Sclerolinon* C. M. Rogers. Blue-flowered flax is also subdivided into groups according to the botanical sections *Stellerolinum* Juz. ex Prob., *Dasylinum* (Planchon) Juz., *Adenolinum* (Reichenb.) Juz. (syn. *Linum perenne* L. group) and *Linum* L. The sect. *Linum* includes the essential cultural species *L. usitatissimum.* Many of the wild flax species are important medicinal or ornamental plants. Also, they are considered as potential donors of economically valuable traits to improve the cultivated species. In the last decade, studies of the genome of cultivated flax *L. usitatissimum* have been intensively conducted [30–43], and the chromosome-scale genome assembly has recently become available [30, 43]. However, the number of studies of the genomes of wild representatives of the genus *Linum* is still very limited. Many wild species have been studied using molecular-karyological approaches [44–48]. For four species, transcriptomes were recently sequenced [49]. Besides, some molecular-phylogenetic studies of the genus *Linum* were carried out [28, 29, 45, 49–54]. In particular, it was shown [28, 54] that the only representative of the sect. *Stellerolinum, L. stelleroides* Planch. (2n = 20), formed the basal phylogenetic branch of the extant blue-flowered flax. After this branch, the phylogenetic branch, represented by modern species of the sect. *Dasylinum* (2n = 16, 32), separated from the common trunk of the phylogenetic tree. Then, the phylogenetic branches corresponding to the sections *Adenolinum* (2n = 18, 36) and *Linum* were separated from each other. Unlike species of most sections of blue-flowered flax, representatives of the sect. *Linum* were not a homogeneous group, and in turn, subdivided into several subgroups of karyologically distinct species. Particularly, *L. narbonense* L. (2n = 4x = 28), 16-chromosome species - *L. grandiflorum* Desf. and *L. decumbens* Desf., 30 chromosome species - *L. usitatissimum* L. and *L. angustifolium* Huds. and also high polyploid species from Australia and New Zealand (*L. marginale* A.Cunn. ex Planch and *L. monogynum* G. Forst. (2n = 84)) represented individual subbranches inside the sect. *Linum*. In the present study, for better understanding of the organization and evolution of the flax genomes, we conducted low-depth sequencing and comparative repeatome study of the representatives of blue-flowered flax using NGS data.

## Materials and methods

### Plant material

Five representatives of the genus *Linum* were selected for genome sequencing. Three of them were obtained from the genebank of Leibniz Institute of Plant Genetics and Crop Plant Research (IPK) (Gatersleben, Germany): *L. hirsutum* subsp. *hirsutum* L. (accession number LIN 1649, (hir)), *L. narbonense* L. (accession number LIN 2002, (nar1)) and *L. usitatissimum* L., var. Stormont cirrus (accession number LIN 2016, (usi)). The sample of *L. perenne* L. (per1) was collected from the natural population (village Nesvetai neighbourhood, Rostov region, Russian Federation) by Dr. A.A. Svetlova, Komarov Botanical Institute RAS (St. Petersburg, Russian Federation). The sample of *L. stelleroides* Planchon (ste) was kindly provided by Dr. L.N. Mironova, Botanic Garden Institute of the Far-Eastern Branch of the Russian Academy of Sciences (Vladivostok, Russian Federation).

For genome size estimation, in addition to the above-listed samples, five species from IPK genebank: *L. leonii* F.W.Schultz (accession number LIN 1672 (leo)), *L. lewisii* Pursh (accession number LIN 1648, (lew)), *L. grandiflorum* Desf. (accession number LIN 974 (gra)), *L.decumbens* Desf. (accession number LIN 1754 (dec)) and *L. angustifolium* Huds. (accession number LIN 1642 (ang)) were used.

### Genome size estimation

The nuclear DNA content of the flax species was determined by comparative Feulgen photometry according to Boyko et al., [55]. Briefly, the modification of this method includes synchronization of cells of root meristems by cold treatment at 0–4^0^ C for night, fixation in ethanol:acetic acid fixative (3:1) at 2–4^0^ C, hydrolysis at 50^0^ C in 1 N HCl for 40 min, maceration with Cellulysin (Calbiochem, USA) and root tips squashing on the microscopic slides. About 50 prophase nuclei have been measured for each flax sample with Opton scanning microphotometer. Measurements were made in relation to to diploid rat hepatocytes containing 7.8 pg DNA per 2C nucleus [56] and *Hordeum vulgare* var. Odesski 31 containing 22.6 pg DNA per 4C nucleus [57].

### DNA extraction, library construction, and sequencing

Flax seedlings were used for DNA extraction as described earlier [50]. High-quality DNA was used for DNA library preparation with TruSeq DNA Sample Prep Kit (Illumina, USA): 1000 ng of each sample was fragmented by nebulization, and then end repair, 3′-end adenylation, and adapter ligation were performed following the manufacturer’s protocol. DNA fragments about 500–700 bp were excised from agarose gel and purified with MinElute Gel Extraction Kit (Qiagen, USA). Enrichment of DNA fragments was performed using PCR Master Mix and Primer Cocktail (Illumina, USA). Quality and concentration of the obtained libraries were evaluated using Agilent 2100 Bioanalyzer (Agilent Technologies, USA) and Qubit 2.0 fluorometer (Life Technologies, USA). The libraries were sequenced on MiSeq sequencer (Illumina, USA), and paired-end reads (300 + 300 nucleotides) were obtained.

### Genomic repetitive fraction identification and analysis

For the analysis of the repeatomes, raw paired-end reads obtained as a result of WGS of the five flax species carried out within the framework of this study and also raw paired-end reads of the WGS of wild flax species available in the European Nucleotide Archive (ENA) [58]: *L. leonii* (accession number SRR1592650, (leo)), *L. lewissii* (accession number SRR1592654, (lew)), *L. perenne* (accession number SRR1592548, (per2)), *L narbonense* (accession number SRR1592545, (nar2)) *L.grandiflorum* (accession number SRR1592647, (gra)), *L. decumbens* (accession number SRR1592610, (dec)) and *L. angustifolium* (accession number SRR1592607, (ang)).

To identify and classify repetitive sequences in flax genomes, raw paired-end WGS reads were analyzed using the RepeatExplorer toolkit [17]. For each studied genome, the WGS reads were filtered by quality, and then they were randomly sampled to final genome coverage about 0.1X, trimmed to 100 bp length and clustered using the graph-based clustering algorithm. A read similarity cut-off of 90% was used for clustering. The reads belonging to the same clusters were assembled into contigs. A minimum sequence overlapping length of 55% was used for assembly. The obtained sequence clusters were identified based on a similarity search against a repeat database implemented in Repeat Explorer. Identification and characterization of tandem repeat sequences were conducted by TAREAN tool of the Repeat Explorer. Clusters containing satellite repeats were identified based on a globular- or ring-like shape of cluster graphs. The monomers reconstruction of satellite repeats were generated using k-mer analysis. We took into consideration only putative satellite sequences having the probability of being a satellite DNA of at least 0.1 and constituting not less than 0.01% of the genome. The obtained putative satellite repeats were compared with known sequences from NCBI by BLASTN.

### Primers design and PCR-amplification of putative satellite DNAs

For all flax samples except *L. usitatissimum* and *L. bienne*, tandem organization of the found putative satDNAs families were additionally examined by PCR amplification. For this purpose, primers were designed in opposite orientation to most conserved regions of monomers consensus sequences (Table 1).

**Table 1:** Primer sequences

STE_sat1	F: 5′- AGTTTCATGTGTCTTAGTGTCTTCT − 3′ R: 5′- AAATTTGAATCCAAGTACACAACCG − 3’
HIR_sat1	F: 5’- GTCTCCGAAAGAGTGTAATG-3′ R: 5′- CTCGTCGCAGAAAAAAGTGTA-3’
HIR _sat2	F: 5’- GCGATTTAGAATAGTTCCGGACCAC-3′ R: 5′- GGTCTAGAATTTGAATCTGGCGAA-3’
HIR _sat3	F: 5’- CCCCTAGGGAAGGCTCCAC-3′ R: 5′- ATCCGGACCGGGTTCCTTTA-3’
HIR _sat4	F: 5’- TCATAGGGGTACCATCACACAA-3′ R: 5′- CGACCTAAATTGAACGTGGCT-3’
LIN_ADE sat	F: 5’- GGAACGGGTGATAAGTCAAGC-3′ R: 5′- TGCGATCCATTCTGACCCTG-3’
ADE _sat1	F: 5’- TAAAACTAAATTCGTAACCGGT-3′ R: 5′- TGGTTTAAGCAGTAGTACGAA-3’
ADE _sat2	F: 5’- GCTAACCATCACCGAAATGGC-3′ R: 5′- AATTGGAAAGTACTCAAAATGGCT-3’
ADE _sat3	F: 5’- AACTCCACTGCACCAAAGC-3′ R: 5′- TCCTACATTGAAGAAGACTTTACCT-3’
ADE _sat4	F: 5’- ATATTTGTCATCCATGTTTTCCCAC-3′ R: 5′- TAAAGGGAGTCCTAGAAGAGAACA-3’
ADE _sat5	F: 5’- TTTGGCTTAAACGCCTAGATTTCC-3′ R: 5′- ATCTTCAATAATTGGCCATTTCGGT-3’
NAR_sat1	F: 5’- GGTATGATCGCACCCGTCTA-3′ R: 5′- AGCTCGGAGAAAGGAGAGAC-3’
NAR _sat2	F: 5’- TGTGAGTTCCATTATGTCATCCCC-3′ R: 5′- TTCTAGTCGTTACCAACACTCACG-3’
16-CH_ sat1	F: 5’- GTCACCGGGCCTGTTTT-3′ R: 5′- TGTTATTTGGCTCGAAACTGAG-3’
16-CH _ sat2	F: 5’- TGATGAATGATAGGATCTACAAGGAGGGC-3′ R: 5′- TGGGAACGCAGAAAAGATGAGAGTTTG-3’
16-CH _ sat3	F: 5’- CCCGGCATAGTCAAACGGC-3′ R: 5′- CGAAAAGAGCATAAAAAGTGGATGG-3’
16-CH _ sat4	F: 5’- GGACAAATTTCACGTTAAGTGGTCCAAG-3′ R: 5′- TGATGGAACAAATGAATTATGAATTGGAGTCTT-3’

STE – *L. stelleroides*; HIR – *L. hirsutum*; LIN_ADE - members of sect. *Adenolinum* and *Linum*; ADE –members of sect. *Adenolinum*; NAR - *L. narbonense*; 16-CH - 16-chromosomal species of sect*. Linum*

The characteristic ladder pattern of tandem repeats was checked after electrophoresis in a 2% agarose gel. The reliability of the PCR product was confirmed by Sanger sequencing. For *L. usitatissimum* and *L. angustifolium,* the reliability of putative satDNA families were verified by BLASTN comparison with a BioNano genome (BNG) optical map of *L. usitatissimum* cv. CDC Bethune [59] available in GenBank (NCBI) under GenomeProject ID #68161 (accession numbers CP027619 - CP027633).

### Phylogenetic analysis and statistical evaluation

For phylogenetic tree construction based on repeatome compositions, the previously published approach has been used [60]. Clusters corresponding to plastid and mitochondrial DNA sequences were filtered out prior to phylogenetic inference. Each abundance was divided by the correcting factor (largest abundance/65) to make all numbers in the matrices ≤ 65 as it is required for TNT tree searches [61, 62]. Data matrix containing relative proportion of top 200 the most abundant clusters was converted to the TNT format (modified Hennig86). Resampling was performed using 100 replicates. The tree was visualized by the iTOL tool [63].

## Results

### General characteristics of genomes

The analysis of genome size and repeatomes showed that species from different sections differed significantly in genome size and repeatome composition (Table 2). So, the only representative of the sect. *Stellerolinum*, diploid species *L. stelleroides*, had the largest genome (1C = 1376 M bp), 39.47% of which was represented by mobile elements, and only 0.55% of satellite DNA. The genome of *L. hirsutum* from sect. *Dasylinum* (1C = 1066 M bp) contained 34.52% of TEs and 2.27% of satellite DNA. Genomes of diploid species of sect. *Adenolinum* were similar in size and repeatome composition. They had an average genome size 446 M bp, TEs - 29.57%, unclassified dispersed repeats - 4.46% and satellite DNA ~ 4.67%. Unlike previous sect. *Linum* united, several groups of species differed significantly in the structure of their karyotypes. We investigated representatives of three such karyo-groups, which were most widely represented in the European flora. It was found that, together with karyological differences, representatives of these three groups also differed significantly in the molecular organization of their genomes. Among them, the group represented by the autotetraploid species *L. narbonense* (2n = 4x = 28) had the largest genome size 1C = 1617 M bp (or 808 M bp per one subgenome). Dispersed repeats represented a significant portion of its genome (54.78%), and the proportion of satellite DNAs (0.87%) was smaller than in the representatives of the previous sections. The second group of sect. *Linum* constituted two karyologically similar diploid species, *L. grandiflorum* and *L. decumbens* (2n = 16). Their genomes were also very similar (1C ~ 480 Mbp), and contained ~ 25% of dispersed repeats and ~ 10% of satellite DNAs. The third group included karyologically similar allotetraploids (2n = 30): the cultivated species *L. usitatissimum* and its wild ancestor *L. angustifolium*. Both had the smallest genomes (1C ~ 330 M bp, an average of 165 M bp per one subgenome), ~ 18% of dispersed repeats and ~ 13% of satellite DNAs.

**Table 2:** Percentage of different families of repeated sequences in the genomes of blue-flowered flax

Repeated sequences	STE	HIR	ADE	NAR	16-CH	30-CH
Satellite	0.55	2.02	4.36	0.87	9.66	12.69
Mobile_element						
Class_I						
LTR						
Ty1_copia		0.02		0.02		
Ale					0.16	0.04
Angela	3.64	0.05	0.13	0.34		
Bianca	0.08				0.01	0.78
Ivana		0.03	0.02			0.22
SIRE	0.12	0.14				
TAR	0.19	0.01	0.03		0.38	0.36
Tork		0.40		0.18	0.02	0.17
Ty3_gypsy						
non-chromovirus						
Athila	17.73	25.39	20.69	12.17	19.84	0.63
TatIV_Ogre	3.11	0.51	0.01	39.11	0.03	
TatV	0.87	0.89	0.66			
chromovirus						
CRM	0.32	0.33	2.61		0.16	
Tekay	15.84	1.05	0.40	0.88	3.51	
pararetrovirus			0.01			
LINE		0.04				
Class_II						
Subclass_1						
TIR						
EnSpm_CACTA	1.28	1.60	0.36	0.08	0.28	
MuDR_Mutator		0.40	0.17	0.02	0.05	0.14
Mobile_element (total)	39.47	34.52	24.39	52.89	24.78	2.34
Unclassified_repeats			4.67	2.97	2.15	15.63
Genome size (bp × 10^9^/1C)	1.38 ± 0.18	1.07 ± 0.12	0.45 ± 0.03	1.61 ± 0.16	0.48 ± 0.06	0.33 ± 0.04

STE – *L. stelleroides*; HIR – *L. hirsutum*; ADE – mean values for members of sect. *Adenolinum*; NAR - *L. narbonense*; 16-CH - mean values for 16-chromosomal members of sect*. Linum*; 30-CH - mean values for 30-chromosomal members of sect. *Linum*

### Dispersed repeats

Comparison of the repeatomes of the studied species showed that they all contained spectra of TE similar in composition (Table 2). In blue flax, Ty3-gypsy LRT elements were most widely represented. Among them, the most common was Athila. Its amount in the genomes of different species varied from 25% for the *L. hirsutum* to 0.6% for the 30-chromosome species of sect. *Linum*. In *L. stelleroides*, together with Ty3-gypsy LRT-element Athila, Ty3-gypsy LRT-element Tekai also was widely represented (~ 16%), while the genome of *L. narbonense* contained considerable amount (~ 39%) of Ty3-gypsy LRT-element Ogre. In contrary, the genomes of other species contain minimal amounts of Ty3-gypsy LRT-element Ogre. Amplification of Ty3-gypsy LRT-element Tekai in *L. stelleroides* and Ty3-gypsy LRT-element Ogre in *L. narbonense* resulted in increasing of the genome sizes of these species.

### Satellite DNA

In total, 44 families of tandem-organized repeats were identified in the genomes of the studied species. Our results showed that the satellite DNA of blue-flower flax evolved much faster than dispersed repeats. Common families of satellite DNAs were only found within the three most closely related groups of species that had similar karyotypes (representatives of sect. *Adenolinum*, 16- and 30-chromosome species of sect. *Linum*). Only one family of putative satellite repeat common to sect. *Adenolinum* and sect. *Linum* was identified using the Repeat Explorer. However, this putative satellite had low confidence, and PCR amplification did not confirm its tandem organization. For other karyologically different species, strictly specific sets of families of satellite DNAs were identified. At the same time, DNA sequences homologous to satellite repeats of one species were often found in genomes of other non-closely related species but in a low number of copies (Additional file 1). The genomes of different species also differed in the content and diversity of satellite DNA (Table 2 and Additional file 1). For instance, in *L. stelleroides*, only one family of a putative satellite was detected with low confidence which made up about 0.55% of its genome. After PCR amplification, only very weak bands corresponding to the monomers and dimers of this repeat were detected. In our opinion, the question of whether putative satellite of *L. stelleroides* really has a tandem organization needs further investigation. In the *L. hirsutum* genome, four families of satellite DNAs were detected and confirmed by PCR. Their total content was 2.3% of the genome. In the genomes of species of sect. *Adenolinum*, the presence of five families of satellites was confirmed. Their total content and the abundance of each of the families varied between species (mean value was 4.4%). In *L. narbonense,* only two families of satellite DNA were discovered which totally made up ~ 0.9% of the overall genome. In 16-chromosomal species of sect. *Linum*, two families of satellite DNAs common to both *L. grandiflorum* and *L. decumbens* were identified. One more family was found only in *L. grandiflorum*, and one was specific to *L. decumbens*. The highest number of families of satellite repeats (28 families) was found in 30-chromosomal species of the sect. *Linum*. In these species as well as in representatives of sect. *Adenolinum*, the amount of each satellite DNA family varied between *L. usitatissimum* and *L. angustifolium*.

**Fig. 1:**
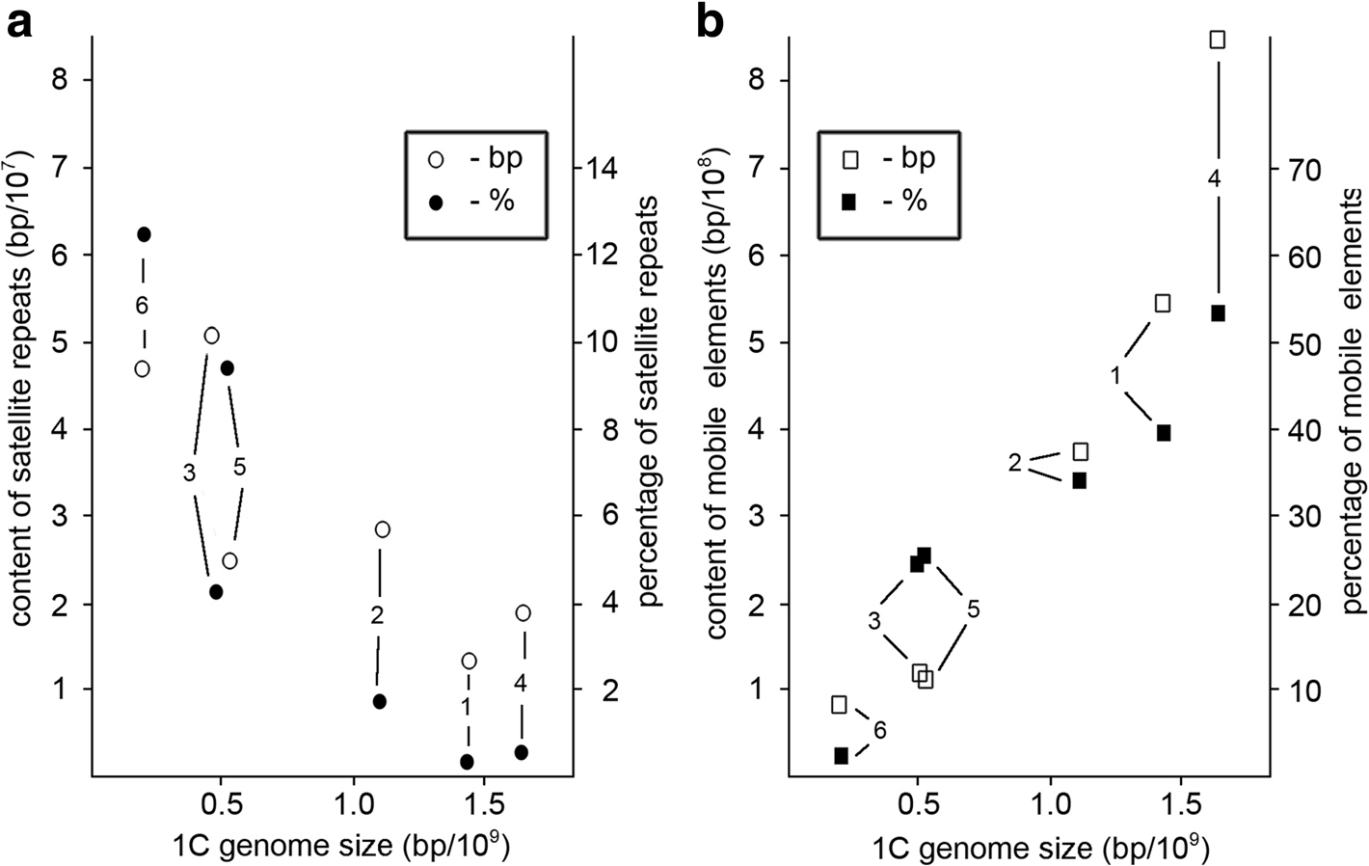
The relationship between 1C DNA amount and the content of repeated sequences (**a** - satellite DNA; **b -** mobile elements) in the studied genomes. 1 - *L. stelleroides* (sect. *Stellerolinum)*; 2 - *L. hirsutum* (sect. *Dasyllinum)*; 3 – mean value for members of the sect. *Adenolinum*; 4 - *L. narbonense* (sect. *Linum*); 5 – mean value for 16-chromosomal species of sect. *Linum*; 6 - mean value for 30-chromosomal species of sect. *Linum*

Thus, the studied species had a distinct tendency to increase the total number and diversity of satellite repeats simultaneous with the decreasing the content of dispersed repeats and the size of the genome (Fig. 1).

### Phylogeny

**Fig. 2:**
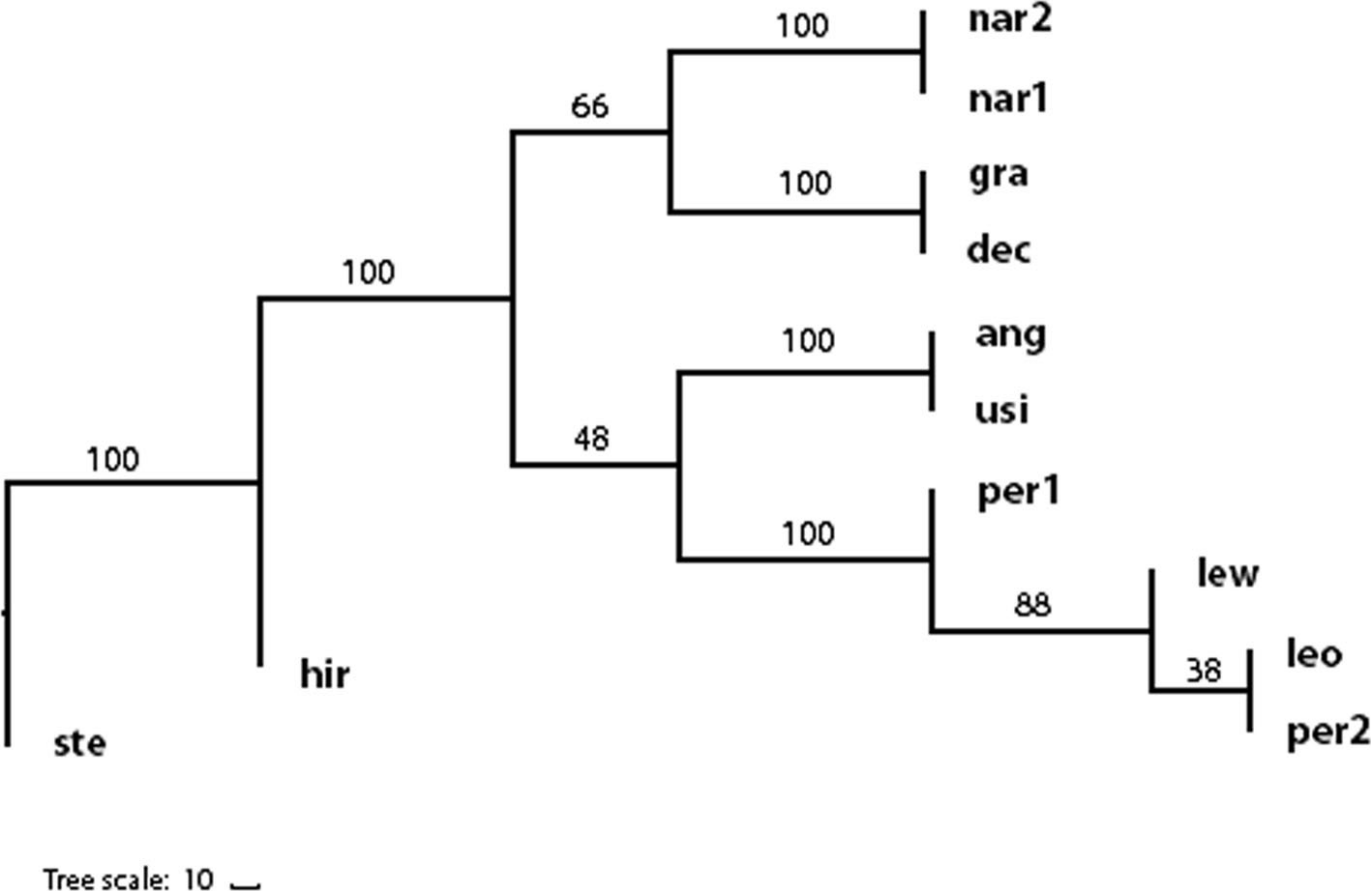
Repeat tree based on 200 top RepeatExplorer clusters. Numbers on nodes represent bootstarp values. Ste - *L. stelleroides*; hir - *L. hirsutum*; per1, per2 - two samples of *L. perenne*; leo - *L. leonii*; lew - *L. lewisii*; nar1, nar2 - two samples of *L. narbonense*; gra - *L. grandiflorum*; dec - *L.decumbens*; ang - *L. angustifolium*; usi - *L. usitatissimum*

Based on the comparison of the repeatomes, a phylogenetic tree of blue-flowered flax was constructed (Fig. 2). It was found that *L. stelleroides* and *L. hirsutum* were the most phylogenetically remote from the rest of the species. The representatives of sect. *Adenolinum* formed one common cluster, and the members of the sect. *Linum* formed three separate subclusters which corresponded to *L. narbonense*, 16- and 30-chromosome species.

## Discussion

### Phylogenetic connections in the clade of blue-flowered flax

The phylogenetic relationships of representatives of the genus *Linum* have been investigated using various molecular phylogenetic markers, including different methods of genomic fingerprinting, and also by comparison of a large number of coding sequences from cytoplasmic and nuclear genomes [28, 29, 45, 49–54]. Besides, for most taxa of the *Linaceae* family, the origin time was estimated [28, 29, 53]. It was shown that the genus *Linum* comprised the representatives of two related clades: yellow-flowered flax and blue-flowered flax. Both clades were subdivided into a number of subclusters. In particular, the blue-flower clade was subdivided into seven subclusters. The two basal ones were formed by representatives of the botanical sections *Stellerolinum* and *Dasylinuym*, respectively. Then the trunk of the phylogenetic tree was subdivided into two branches which corresponded to the members of the sections *Adenolinum* and *Linum*. The branch represented by the species of sect. *Linum* was splitted into four subclusters. The first three of them included *L. narbonense*, 16-chromosome species (*L.grandiflorum* and *L. decumbens*) and 30-chromosome species (*L.angustifolium* and *L. usitatissimum*), respectively. The fourth cluster was represented by highly polyploid and closely related species from Australia and New Zealand (*L. marginale* A.Cunn. ex Planch and *L. monogynum* G. Forst.) which were not examined in this study.

Thus, the reconstructed here phylogeny of blue-flowered flax, which was based on the similarities and differences of the repeatomes, agreed with the results of molecular phylogenetic studies reported earlier.

### Interspecies variability of dispersed repeats

As in the case of other angiosperms [64–66], genome size in flax species was mainly determined by the content of repeated sequences, especially by the dispersed repeats. The data on the size and composition of the genomes in the studied group of flax were in a good agreement with the results of the karyological investigations performed earlier [45–47, 54]. Particularly, the species with the largest genome size (*L. stelleroides, L. hirsutum* and *L. narbonense*) had the largest chromosome sizes, whereas 30- chromosome species of the sect. *Linum* with the smallest chromosome sizes had the smallest genomes.

We revealed that all the studied genomes were characterized by similar sets of transposable elements. Among them, LTR type retroelements were the most widely represented. It was probably due to the common origin of those genomes. At the same time, differences were found both in the total content of dispersed repeats and in the content of individual classes of mobile elements in different species. Like other plants, in the studied species there is a direct relationship between the content of dispersed repeats in the genome and its size. Thus, the extremely large genome sizes in *L. stelleroides*, *L. hirsutum*, and *L. narbonense* could be related to a significant amplification of certain mobile elements. The smallest genome sizes were found in 30-chromosomal species of sect. *Linum* (*L. usitatissimum* and *L. angustifolium*) which also contained the lowest amount of dispersed repeats (20 and 18%, respectively). It should be noted that the content of dispersed repeats in *L. usitatissimum*, determined by using Repeat Explorer, was lower than that obtained earlier (24%) [30, 59]. It could be related to the genomic differences among the investigated flax varieties or/and the smaller accuracy of the estimation method applied here. The small genome sizes of the 30-chromosomal allotetraploid *Linum* species [59] could be a result of the genome downsizing occurring after the formation of allotetraploids that was described for many plant species [64–69].

### Interspecific variability of satellite DNAs

It is known that satellite DNA families is often retained in related species during long evolutionary periods. In particular, in some plant groups, there are families of satellite DNAs specific to taxa of different rank up to families and orders [70–73]. However, satellite DNAs sometimes evolve very rapidly and can differ considerably even in closely related species [74]. It is believed that the rapid divergence of satellite sequences occurs in reproductively isolated populations through a mechanism of concerted evolution [75–77]. In blue-flowered flax, the karyologically distinct species had different sequences of satellite DNAs, whereas closely related species with similar karyotypes had similar satellite DNAs. At the same time, we found that the content and diversity of satellite DNAs could differ even in closely related species and in different samples of the same species. The most significant differences in sets of satellite repeats were found in phylogenetically distant species. There was a tendency to increase the total content and the number of families of satellite DNA with decreasing genome size. We revealed that the sequences homologous to satellite DNAs of certain species could be also found in the genomes of phylogenetically distant species but in low copy numbers and these findings were in a good agreement with the ‘library’ hypothesis [78]. According to this hypothesis, related species shared a library of ancestral repeated sequences in low copy numbers. Some of these sequences could be differentially amplified creating a satellite DNAs of the particular species. It is suggested that a change in the amount of satellite DNAs might appear as a result of unequal crossover, chromosomal translocations, segmental chromosomal duplications or deletions as well as rolling-circle replication of extrachromosomal circular DNAs and their reinsertion in chromosomes [77, 79, 80]. The opposite directions of changes observed in the amount of tandem and dispersed repeats during evolution of blue-flowered flax were probably not accidental. Both types of repeated sequences were predominantly located in the heterochromatic regions of the chromosomes, where they were often interspersed with each other [81–83]. Currently, many data have been indicated that mobile elements can influence the evolution of satellite DNAs participating in the formation of new families of tandem repeats and their distribution along the genomes [84–88].

## Conclusions

The obtained results showed that the evolution of the blue-flowered *Linum* species was accompanied by waves of amplification of satellite DNAs and LTR retrotransposons. Those events together with polyploidization resulted in significant differences in genome size and karyotype structure of blue-flowered flax. The genomes of the studied flax species contained similar sets of mobile elements but differed in their amount. At the same time, comparison of satellite DNAs showed that most of the detected tandem repeats were species-specific (except for some very closely related species) which indicated the rapid and concerted evolution of this genome fraction. The phylogenetic relationships between the studied flax species, obtained by the estimation of the similarity of their repeatomes, agreed with the previous data based on other phylogenetic markers. A direct relationship between the sizes of the genomes and the total content of dispersed repeats and the inverse relationship between the sizes of the genomes and the total content and diversity of satellite DNAs were revealed. The lowest amount of dispersed repeats and highest content of satellite DNAs in the genomes of 30-chromosome flax from the sect. *Linum* was probably a result of their allotetraploid origin. Thus, our findings gave new useful information about *Linum* genome evolution and provided a valuable set of data that could be used in future investigation of *Linum* genome.

## Additional file

Additional file 1: Putative satellite DNA sequences in genomes of blue-flowered flax. (DOCX 1037 kb)

## References

[1] López-Flores I, Garrido-Ramos MA (2012). The repetitive DNA content of eukaryotic genomes. Genome Dyn.

[2] Elder JF, Turner BJ (1995). Concerted evolution of repetitive DNA sequence in eukaryotes. Q Rev Biol.

[3] Kubis SE, Schmidt T, Heslop-Harrison JS (1998). Repetitive DNA elements as a major component of plant genomes. Ann Bot.

[4] Ugarković D, Plohl M (2002). New EMBO member’s review: variation in satellite DNA profiles—causes and effects. EMBO J.

[5] Schmidt AL, Anderson LM (2006). Repetitive DNA elements as mediators of genomic change in response to environmental cues. Biol Rev.

[6] Biemont C, Vieira C (2006). Junk DNA as an evolutionary force. Nature.

[7] Oliver KR, Greene WK (2009). Transposable elements: powerful facilitators of evolution. BioEssays.

[8] Hua-Van A, Boutin A, Le Rouzic TS, Filée J, Capy P (2011). The struggle for life of the genome's selfish architects. Biol Direct.

[9] Chénais B, Caruso A, Hiard S, Casse N (2012). The impact of transposable elements on eukaryotic genomes: from genome size increase to genetic adaptation to stressful environments. Gene.

[10] Tolli M, Boissinot S (2012). The evolutionary dynamics of transposable elements in eukaryote genomes. Genome Dyn..

[11] Ferree PM, Barbash DA (2009). Species-specific heterochromatin prevents mitotic chromosome segregation causing hybrid lethality in *Drosophila*. PLoS Biol.

[12] Grewal SI, Elgin SC (2007). Transcription and RNA interference in the formation of heterochromatin. Nature.

[13] Meštrović N, Plohl M, Mravinac B, Ugarković D (1998). Evolution of satellite DNAs from the genus *Palorus* - experimental evidence for the ‘library’ hypothesis. Mol Biol Evol.

[14] Malik HS, Henikoff S (2001). Adaptive evolution of Cid, a centromere-specific histone in *Drosophila*. Genetics.

[15] Navajas-Perez R, Paterson AH (2009). Patterns of tandem repetition in plant whole genome assemblies. Mol Gen Genomics.

[16] Novák P, Neumann P, Macas J (2010). Graph-based clustering and characterization of repetitive sequences in next-generation sequencing data. BMC Bioinformatics.

[17] Novák P, Neumann P, Pech J, Steinhaisl J, Macas J (2013). Repeat explorer: a galaxy-based web server for genome-wide characterization of eukaryotic repetitive elements from next-generation sequence reads. Bioinformatics.

[18] Macas J, Kejnovský E, Neumann P, Novák P, Koblížková A, Vyskot B (2011). Next generation sequencing-based analysis of repetitive DNA in the model dioecious [corrected] plant Silene latifolia. PLoS One.

[19] González LG, Deyholos MK (2012). Identification, characterization and distribution of transposable elements in the flax (Linum usitatissimum L.) genome. BMC Genomics.

[20] Staton SE, Bakken BH, Blackman BK, Chapman MA, Kane NC, Tang S (2012). The sunflower (*Helianthus annuus* L.) genome reflects a recent history of biased accumulation of transposable elements. Plant J.

[21] Steflova P, Tokan V, Vogel I, Lexa M, Macas J, Novak P (2013). Et all. Contrasting patterns of transposable element and satellite distribution on sex chromosomes (XY1Y2) in the dioecious plant Rumex acetosa. Genome Biol Evol.

[22] Sveinsson S, Gill N, Kane NC, Cronk Q (2013). Transposon fingerprinting using low coverage whole genome shotgun sequencing in cacao (*Theobroma cacao* L.) and related species. BMC Genomics.

[23] Novák P, Hřibová E, Neumann P, Koblížková A, Doležel J, Macas J (2014). Genome-wide analysis of repeat diversity across the family Musaceae. PLoS One.

[24] Barghini E, Natali L, Cossu RM, Giordani T, Pindo M, Cattonaro F (2014). Et all. The peculiar landscape of repetitive sequences in the olive (Olea europaea L.) genome. Genome Biol Evol..

[25] Macas J, Novák P, Pellicer J, Čížková J, Koblížková A, Neumann P (2015). In depth characterization of repetitive DNA in 23 plant genomes reveals sources of genome size variation in the legume tribe Fabeae. PLoS One.

[26] Kirov IV, Kiseleva AV, Laere KV, Roy NV, Khrustaleva LI (2017). Tandem repeats of *Allium fistulosum* associated with major chromosomal landmarks. Mol Genet Genomics.

[27] González ML, Chiapella JO, Urdampilleta JD (2018). Characterization of some satellite DNA families in *Deschampsia Antarctica* (Poaceae). Polar Biol.

[28] McDill J, Repplinger M, Simpson BB, Kadereit JW (2009). The phylogeny of Linum and Linaceae subfamily Linoideae, with implications for their systematics, biogeography, and evolution of heterostyly. Syst Bot.

[29] McDill J, Simpson BB (2011). Molecular phylogenetics of Linaceae with complete generic sampling and data from two plastid genes. Bot J Linn Soc.

[30] Wang Z, Hobson N, Galindo L, Zhu S, Shi D, McDill J (2012). The genome of flax (Linum usitatissimum) assembled de novo from short shotgun sequence reads. Plant J.

[31] Johnson C, Moss T, Cullis C (2011). Environmentally induced heritable changes in flax. J Vis Exp.

[32] Fu YB, Peterson GW (2012). Developing genomic resources in two Linum species via 454 pyrosequencing and genomic reduction. Mol Ecol Resour.

[33] Melnikova NV, Dmitriev AA, Belenikin MS, Koroban NV, Speranskaya AS, Krinitsina AA (2016). Identification, expression analysis, and target prediction of flax genotroph microRNAs under normal and nutrient stress conditions. Front Plant Sci.

[34] Zhang N, Deyholos MK (2016). RNAseq analysis of the shoot apex of flax (*Linum usitatissimum*) to identify phloem fiber specification genes. Front Plant Sci.

[35] Kumar S, You FM, Cloutier S (2012). Genome wide SNP discovery in flax through next generation sequencing of reduced representation libraries. BMC Genomics.

[36] Dmitriev AA, Kudryavtseva AV, Krasnov GS, Koroban NV, Speranskaya AS, Krinitsina AA (2016). Gene expression profiling of flax (*Linum usitatissimum* L.) under edaphic stress. BMC Plant Biol.

[37] Dmitriev AA, Krasnov GS, Rozhmina TA, Kishlyan NV, Zyablitsin AV, Sadritdinova AF (2016). Glutathione S-transferases and UDP-glycosyltransferases are involved in response to aluminum stress in flax. Front Plant Sci.

[38] Dash PK, Cao Y, Jailani AK, Gupta P, Venglat P, Xiang D (2014). Genome-wide analysis of drought induced gene expression changes in flax (Linum usitatissimum). GM Crops Food.

[39] Yu Y, Wu G, Yuan H, Cheng L, Zhao D, Huang W (2016). Identification and characterization of miRNAs and targets in flax (Linum usitatissimum) under saline, alkaline, and saline-alkaline stresses. BMC Plant Biol.

[40] Galindo-Gonzalez L, Pinzon-Latorre D, Bergen EA, Jensen DC, Deyholos MK (2015). Ion torrent sequencing as a tool for mutation discovery in the flax (Linum usitatissimum L.) genome. Plant Methods.

[41] Dmitriev AA, Kudryavtseva AV, Bolsheva NL, et al. miR319, miR390, and miR393 Are Involved in Aluminum Response in Flax (*Linum usitatissimum* L.). BioMed Research International. 2017;4975146. 10.1155/2017/4975146. 10.1155/2017/4975146 PMC5337325 28299328

[42] Dmitriev AA, Krasnov GS, Rozhmina TA, Novakovskiy RO, Snezhkina AV, Fedorova MS, et al. Differential gene expression in response to *Fusarium oxysporum* infection in resistant and susceptible genotypes of flax (*Linum usitatissimum* L.). BMC Plant Biol. 2017;(17 Suppl 2):253. 10.1186/s12870-017-1192-2 PMC5751779 29297347

[43] Yi L, Gao F, Siqin B, Zhou Y, Li Q, Zhao X, et al. Construction of an SNP-based high-density linkage map for flax (*Linum usitatissimum* L.) using specific length amplified fragment sequencing (SLAF-seq) technology. Kulwal PL, editor. PLoS One. 2017;12(12):e0189785. 10.1371/journal.pone.0189785 PMC5739455 29267332

[44] Bolsheva NL, Semenova OY, Muravenko OV, Nosova IV, Popov KV, Zelenin AV (2005). Localization of telomere sequences in chromosomes of two flax species. Biol Membrany.

[45] Muravenko OV, Yurkevich OY, Bolsheva NL, Samatadze TE, Nosova IV, Zelenina DA (2009). Comparison of genomes of eight species of sections Linum and Adenolinum from the genus Linum based on chromosome banding, molecular markers and RAPD analysis. Genetica.

[46] Muravenko OV, Bolsheva NL, Yurkevich OY, Nosova IV, Rachinskaya OA, Samatadze TE (2010). Karyogenomics of species of the genus Linum L. Russ J Genet.

[47] Yurkevich OY, Naumenko-Svetlova AA, Bolsheva NL, Samatadze TE, Rachinskaya OA, Kudryavtseva AV (2013). Investigation of genome polymorphism and seed coat anatomy of species of section Adenolinum from the genus Linum. Genet Resour Crop Ev.

[48] Bolsheva NL, Zelenin AV, Nosova IV, Amosova AV, Samatadze TE, Yurkevich OY (2015). The diversity of karyotypes and genomes within section Syllinum of the genus Linum (Linaceae) revealed by molecular cytogenetic markers and RAPD analysis. PLoS One.

[49] Sveinsson S, McDill J, Wong GK, Li J, Li X, Deyholos MK (2014). Phylogenetic pinpointing of a paleopolyploidy event within the flax genus (Linum) using transcriptomics. Ann Bot.

[50] Vromans J (2006). Molecular genetic studies in flax (*Linum usitatissimum* L.).

[51] Melnikova NV, Kudryavtseva AV, Zelenin AV, Lakunina VA, Yurkevich OY, Speranskaya AS (2014). Retrotransposon-based molecular markers for analysis of genetic diversity within the genus Linum. Biomed Res.

[52] Fu YB, Dong Y, Yang MH (2016). Multiplexed shotgun sequencing reveals congruent three-genome phylogenetic signals for four botanical sections of the flax genus Linum. Mol Phylogenet Evol.

[53] Schneider AC, Freyman WA, Guilliams CM, Springer YP, Baldwin BG (2016). Pleistocene radiation of the serpentine-adapted genus *Hesperolinon* and other divergence times in Linaceae (Malpighiales). Am J Bot.

[54] Bolsheva NL, Melnikova NV, Kirov IV, Speranskaya AS, Krinitsina AA, Dmitriev AA, et al. Evolution of blue-flowered species of genus Linum based on high-throughput sequencing of ribosomal RNA genes. BMC Evol Biol. 2017;(17Suppl 2):253. 10.1186/s12862-017-1105-x PMC5751768 29297314

[55] Boyko EV, Badaev NS, Maximov NG, Zelenin AV (1984). Does DNA content change in the course of Triticale breeding?. Cereal Research Communication.

[56] Edstrom J-E, Kawiak J (1961). Microchemical deoxyribonucleic acid determination in individual cells. J Biophys Biochem Cytol.

[57] Sivolap YM, Sytnik KM (1988). Features of the organization and variability of the genomes of agricultural plants. The genome of plants.

[58] European Nucleotide Archive (ENA). https://www.ebi.ac.uk/ena. Accessed 26 Jan 2019.

[59] You FM, Xiao J, Li P, Yao Z, Jia G, He L, et al. Chromosome-scale pseudomolecules refined by optical, physical and genetic maps in flax. Plant J. 2018. 10.1111/tpj.13944. 10.1111/tpj.13944 29681136

[60] Dodsworth S, Chase MW, Kelly LJ, Leitch IJ, Macas J, Nová P (2014). Genomic repeat abundances contain phylogenetic signal. Syst Biol.

[61] Goloboff PA, Mattoni CI (2006). Continuous characters analyzed as such. Cladistics.

[62] Goloboff PA, Farris JS, Nixon KC (2008). TNT, a free program for phylogenetic analysis. Cladistics.

[63] Letunic I, Bork P (2016). Interactive tree of life (iTOL) v3: an online tool for the display and annotation of phylogenetic and other trees. Nucleic Acids Res.

[64] Boyko EV, Badaev NS, Maximov NG, Zelenin AV (1988). Regularities of genome formation and organization in cereals. I. DNA quantitative changes in the process of allopolyploidization. Russ J Genet.

[65] Heslop-Harrison JS (2000). Comparative genome Organization in Plants: from sequence and markers to chromatin and chromosomes. Plant Cell.

[66] Leitch IJ, Bennett MD (2004). Genome downsizing in polyploid plants. Biol J Linnean Soc.

[67] Eilam T, Anikster Y, Millet E, Manisterski J, Feldman M (2010). Genome size in diploids, allopolyploids, and Autopolyploids of Mediterranean Triticeae. Journal of Botany.

[68] Mehrotra S, Goyal V (2014). Repetitive sequences in plant nuclear DNA: types, distribution. Evolution and Function Genomics Proteomics Bioinformatics.

[69] Li SF, Su T, Cheng GQ, Wang BX, Li X, Deng CL (2017). Chromosome evolution in connection with repetitive sequences and epigenetics in plants. Genes.

[70] Quesada del Bosque ME, López-Flores I, Suárez-Santiago VN, Garrido-Ramos MA (2013). Differential spreading of HinfI satellite DNA variants during radiation in Centaureinae. Ann Bot.

[71] Quesada del Bosque ME, López-Flores I, Suárez-Santiago VN, Garrido-Ramos MA (2014). Satellite-DNA diversification and the evolution of major lineages in Cardueae (Carduoideae, Asteraceae). J Plant Res.

[72] Cafasso D, Chinali G (2014). An ancient satellite DNA has maintained repetitive units of the original structure in most species of the living fossil plant genus *Zamia*. Genome.

[73] Mehrotra S, Goel S, Raina SN, Rajpal VR (2014). Significance of satellite DNA revealed by conservation of a widespread repeat DNA sequence among angiosperms. Appl Biochem Biotechnol.

[74] Macas J, Neumann P, Novák P, Jiang J (2010). Global sequence characterization of rice centromeric satellite based on oligomer frequency analysis in large-scale sequencing data. Bioinformatics.

[75] Dover G (1986). Molecular drive in multigene families: how biological novelties arise, spread and are assimilated. Trends Genet.

[76] Pérez-Gutiérrez MA, Suárez-Santiago VN, López-Flores I, Romero AT, Garrido-Ramos MA (2012). Concerted evolution of satellite DNA in *Sarcocapnos*: a matter of time. Plant Mol Biol.

[77] Plohl M, Meštrović N, Mravinac B, Garrido-Ramos MA (2012). Satellite DNA evolution. Repetitive DNA.

[78] Fry K, Salser W (1977). Nucleotide sequences of HSa satellite DNA from kangaroo rat *Dipodomys ordii* and characterization of similar sequences in other rodents. Cell.

[79] Ma J, Jackson SA (2006). Retrotransposon accumulation and satellite amplification mediated by segmental duplication facilitate centromere expansion in rice. Genome Res.

[80] Navrátilová A, Koblizková A, Macas J (2008). Survey of extrachromosomal circular DNA derived from plant satellite repeats. BMC Plant Biol.

[81] Schueler MG, Higgins AW, Rudd MK, Gustashaw K, Willard HF (2001). Genomic and genetic definition of a functional human сentromere. Science.

[82] Heslop-Harrison JSP, Schwarzacher T (2011). Organisation of the plant genome in chromosomes. Plant J.

[83] Neumann P, Navrátilová A, Koblížková A, Kejnovský E, Hřibová E, Hobza R (2011). Plant centromeric retrotransposons: a structural and cytogenetic perspective. Mob DNA.

[84] Satović E, Vojvoda Zeljko T, Luchetti A, Mantovani B, Plohl M (2016). Adjacent sequences disclose potential for intra-genomic dispersal of satellite DNA repeats and suggest a complex network with transposable elements. BMC Genomics.

[85] Meštrović N, Mravinac B, Pavlek M, Vojvoda-Zeljko T, Šatović E, Plohl M (2015). Structural and functional liaisons between transposable elements and satellite DNAs. Chromosom Res.

[86] Garrido-Ramos MA. Satellite DNA: An Evolving Topic. Liehr T, editor. Genes. 2017;8(9):230. 10.3390/genes8090230 PMC5615363 28926993

[87] Macas J, Koblizkova A, Navratilova A, Neumann P (2009). Hypervariable 3′ UTR region of plant LTR-retrotransposons as a source of novel satellite repeats. Gene.

[88] Gong Z, Wu Y, Koblízková A, Torres GA, Wang K, Iovene M (2012). Repeatless and repeat-based centromeres in potato: implications for centromere evolution. Plant Cell.

