# Supplementary material for: Characterization of repeated DNA sequences in genomes of blue-flowered flax
Source: BMC Evol Biol. 2019 Feb 26;19(Suppl 1):49. doi: 10.1186/s12862-019-1375-6 (PMC6391757; doi:10.1186/s12862-019-1375-6)
Supplement: Supplementary file 1 — Putative satellite DNA sequences in genomes of blue-flowered flax. (DOCX 1037 kb) [file 12862_2019_1375_MOESM1_ESM.docx]

**Supplementary Table 1.** Putative satellite DNA sequences in genomes of blue-flowered flax.

##

| Name | Species | Length [bp] | Genome proportion [%] | Satelliteprobability | Consensus | Graph layout | Similarity based annotation |
| --- | --- | --- | --- | --- | --- | --- | --- |
| STE sat1 | *L. stelleroides* | 433 | 0.35 | 0.108 | TCTTAGTGTCTTCTGAAAGGAGAAACCCCCAAAATAGAGTCTGATTTAGTTGGAATTGAATTTTAGAGTCTTGTTTCTGTTTTCGTTGAGTTCTTATCGAGTTGTAAACTTGTTTTGGGTTAAAAAGCTTGTGTGTGGCTCAAGAAGAAGAAATAAGCACATTAAAAGATCTAAAAGAGAAAAGGATAAACAACAAAGCAAAACACATCACTAAAACACTTGTGTCAAGTCATAAGGGGGCTATTCGATCCAAGAAAGGCGGTTGTTTTGTGTCAAGTTAACCTTAGAGCATAAGAAACCATCGATCGTGACCTTTTTCAAACCATAAACTCAATACTTGTTATTTCTTTCTTTTGAAACCATACCAAGCATTCTCTAAGTCGGTTGTGTACTTGGATTCAAATTTTGTATTGCTTCTTGATTAAGTTTCATGTG | 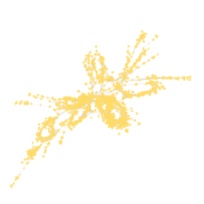 |  |
| HIR sat1 | *L. hirsutum* | 44 | 1.3 | 0.994 | CTCTTTCGGAGACCTCGTCGCAGAAAAAAGTGTAATGCATTACA | 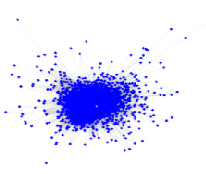 |  |
| HIR sat2 | *L. hirsutum* | 170 | 0.18 | 0.992 | CGAAATTATGACGTTTAAGACTCAATATCTCATTTTTTCGGTTTTGGCCCATTTTCGGTGATGGTACCCCTTTCGGAATTCGCCCGAGAATGAAAGTTGCCAATTTTTTTGCACAGTGGTCCGGAACTATTCTAAATCGCATACATGACAGGTCTAGAATTTGAATCTGG | 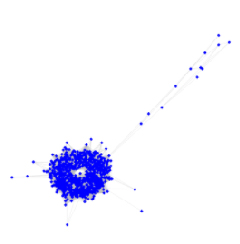 |  |
| HIR sat3 | *L. hirsutum* | 168 | 0.06 | 0.981 | CCCTAGGGGGAATCCGGACCGGGTTCCTTTAGTAATGTTTCTTATCTCATCGAGGAGGACGTGTATATGCGAAATGGGCGCATTCCGGTCAGTTTTCAAGAACCCCAAAATACCCTAAATAGGCTAATGTGGAATCGAAAAGCGGTTTTGCGGAGGCCGTGGAGCCTT | 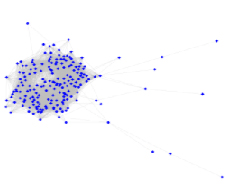 |  |
| HIR sat4 | *L. hirsutum* | 168 | 0.043 | 0.993 | CAAGCATTGAACGGTGGACGAATTCATAAGATTTAGGCTCATTACCGCATTTTTTCGGTTTACTCCATTTTGTGTGATGGTACCCCTATGAAAATCCGACCTAAATTGAACGTGGCTAAATTTTTCAATAGTTGTTTGGAATGATTTGAAATTGAATACAGGACACGT | 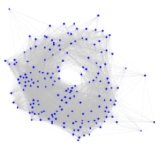 |  |
| ADE - LIN sat | sect. *Adenolinum* and sect. *Linum* | 77 | 0.19 | 0.699 | CAAAATATGCGATCCATTCTGACCCTGGATGCCGAGGTTTTGTGTACTAGGGTCTCAGCTTGACTTATCACCCGTTC | 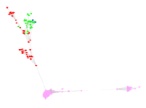 |  |
| ADE sat1 | sect. *Adenolinum* | 743 | 3.12 | 0.992 | CGCACCTAGGATCACGTCATTCAGATATGAATCGTCTATAGCCATGGGCAGATGATCATAATACTATAAGTTGAGTGATCAGGAACAACGAGTCGACCTTAGATTAGACGCAGAGTACTTGGACCCGCTGGTTGTATATAACAAAGAACCCTTAGTGTCCAGAGTAGAATGGTGCCACGAAGAAAAGGGTGATTAACTTGAACAATGATAAACCAAGGCCTAAGAGCTAAGCATTTACATGCTAAAGCTAGAAGGAAGAAAGACAAAAGTTCTGAAATAATAATCTTTAATCAAGGGAGGTTGTGAAACAATGAGTACAATGGATCATATATATATAATTAGATTAATCTTAGGGATTCTCTATGTCCTTCTATGACACTACTACTAATACAATTAAGAATTCATAGCTAAATTAAAAAGGAAGAAACAAACTAAGAGTTCTAAACTAAATAGGAATCGAAATCCGTGTAGATATCTTGTTGTCACTTCAAAATGTCATAACTCCCTTTTCCGGTTGAGTCTAGAAGCGAACAAGGTATGCATGGAAAGAACGTCTCGTTAGCTTTCTAATGGACTTGACCTTGCACCATTTGGGCTTCGGTATCATTAGTTATGTCCGTTTTAGTAAAATGGGGTAGATTGACTTTTCCTCCATCAACAAGGAGAGCTAGTTTCTTTGGGGCCCAAGTAACTTAAGGAGGCTTAGATCCACCTTTTAATCAGAACTTGATGTCTGTTGATGT | 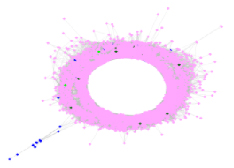 |  |
| ADE sat2 | sect. *Adenolinum* | 51, 102 | 1.70 | 0.974 | TAAATTCGTAACCGGTAGTACGAATTCGTACTAATGCTTAAACGATAAAACTAAATCCCTATTCGGTAGTACAAGTTCGTACTACTGCTTAAACCATAAAAC | 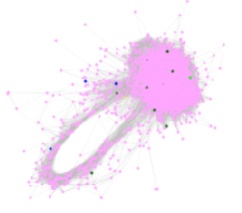 |  |
| ADE sat3 | sect. *Adenolinum* | 168 | 1.40 | 0.989 | GGTTGGCAATATATTTCAGACATTTTACTATCATTCCTGAGAATATTAGCCATTTTGAGTACTTTCCAATTTTTCATAGGCTAACCATCACCGAAATGGCCCAAAACCGACAAATGCGGCATTTTGGCTTAAATACCTAGATTTTCTCCAAATTTTGAGGCGAAACCT | 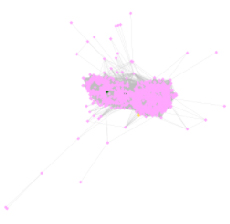 |  |
| ADE sat4 | sect. *Adenolinum* | 287 | 0.91 | 0.981 | ACGTACCAAGGCGTTCCGTAAAATGTAACGAGTTCTAGGTAAAGTCTTCTTCAATGTAGGAAAGCGTAACTCCACTGCACCAAAGCGATCCGTAAAATGTCGTGGGTTCCCAATGAACACTTCTTAACCGAAGGTAAGGCTAATTCCCACTCACCAATGAGTTCCCTATAATACAGCGAGTTTCCGCTAAACTCCCCTTCACCGTAGGAAAGCGTAAGTCCCATGAACAAGGGCATTCCCTAATATGCTATGAACACTCTCTTTTTCTAGAGGGAAACATAACTCCC | 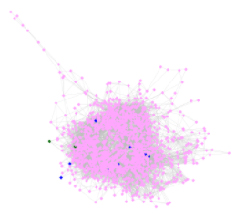 |  |
| ADE sat5 | sect. *Adenolinum* | 153 | 0.560 | 0.992 | CTTCTAGGACTCCCTTTAGACAAATTATATTTGTCATCCATGTTTTCCCACTTTCAAATATCATAATACCGACATTATAGATTATATTCATAAGGAGCAGAAGAGCAACATGAATGACTAAACAATCATCATAGTAGCCATTTAACATGTTCT | 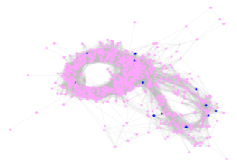 |  |
| NAR sat1 | *L. narbonense* | 109 | 1.7% | 0.905 | AAAGAGGGAAGAAGTAGACGGGTGCGATCATACCAGCTCGGAGAAAGGAGAGACGGCTAATTTTGCTGTTCGGGTAGAAACGGAATAAAATAGTATTAAAACTAAGAAA | 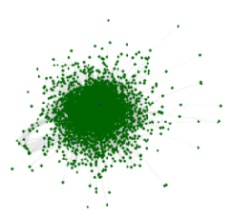 |  |
| NAR sat2 | *L. narbonense* | 162 | 0.83 | 0.981 | GGCTTAGCCATGAAAATTTGAAAATTTTTAAATTGGCTAATTTTTAGCGTGGGTGTTGGTAACGACTAGAAATGCGAGTTCCATTATGTCATCCCTTCAAATTTTAGCCAAATGAAATTGAAATGGCTTTTTTTGTGTTTTTACGATTTTTCGTGTATGTTA | 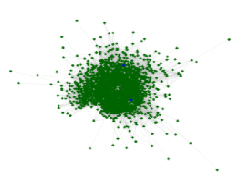 |  |
| 16ch sat1 | *L. grandiflorum,**L. decumbens* | 59, 117 | 9.10 | 0.992 | CAACTCAGTTTCGAGCCAAATAACATTTTTGGTCACCGGGCCTGTTTTGGGTGTTTTTG | 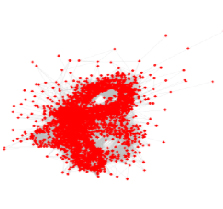 | 0.02% Class_I/LTR/Ty1_copia/Ale:Ty1-RH0.01% organelle/plastid |
| 16ch sat2 | *L. grandiflorum,* | 757 | 0.5 | 0.821 | CATCATTTATTGTTTTGTACCAAACATTGTTAAAGGGGAAGCGATTCACGTAAAAAAATTTACCGTGATTGATGATAGCTGTGTGTCATTATTGTAGTTTGAGAGAACCATGTTGCTTGCAACTTTTGTGTAAGTACATATGATATATGGAACTCCAAAATGGCGAGAGATGAGAAAGAATACACACTAGAACAAGCTATATAATCCAAAAAAGATTCACGAAAATCACAATAAAAACAAAATATAAATCCAAAAAAGATTCAAATGGTATTCCTACAATTTACAAGACAAATTATATTAATCAATAAAGCAAACTCTCATCTTTTCTGCGTTCCCAAAAATTTATTTTGTTGGTGTTTAAAGTACTTTTCTTGCTCAGGAAAAAACTAGTCTTCTTTGATGACTCTTATGATGAATGATAGGATCTACAAGGAGGGCCCATCAGTCTATATATCAATTTATTATTACCTTCACATTTCTCTACATAAACCTCTCTGGGTAATTGGGTTGCCACTTCGAAAAAAGGACTGCTCAAATGCTTTATTCACGTTTCTCTACAAAATGACTGTGCCTTGTAATTGTCACATATCATCAATAAATTTATCTCTCTCTAGGCAGTCCACCAATACCGTGCAATTGGTAGTGGAGCATTGTTCAATTTTTACGTTTGACAACGGCCGGATTGAAATTCGGAGAGTTAATATCTATTTTTCCTTTTTTATTTTTTAGGTTCACTAGTCTATGCTCTATCAAAACC | 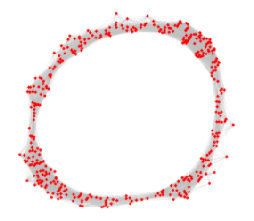 |  |
| 16ch sat3 | *L. grandiflorum,**L. decumbens* | 746 | 0.22 | 0.986 | AGTCAAACGGCCGTAGCTTTGTCCTCGGTTGTCGGAATGCGTTGATGTCTTCTCCGGAACCGAACATATTTCCGAGATCTACGATCTTCGTGAAGGCTTCGCCTCGAGAGGAACTCGTTTTCCCCTTCAAATCGACGCTTTAACGCCCGTTTTTTGCCCGGAACCCGTTTTTTTGGTTTTTCCGACCCGCCCACAGCCTTATCTTCTGGCCGAATGAGTTCATTCTTTTTCCCACGTCGCACGTCTTTCCGAGATCTACGATATTCGTGAAGACTTCGTCGCGAGGGGAGGACGTTCGACCCTTCGAATCGACGTTTTAGTGCCCGTTTTCCTCCGAAATCCACTATTCATGCCCTTTCCCAAACCCACCCAAAAAATCAACTTTTTGCCCGGTTGTCCGATTGAGGTGATTTTTGGCTCTAAATCGCACGTCTTTCCGAGCTCTACGAAATTAGTAAAGGCTCCGTCGCCAAAGGAGGTCGTTTGCCCCTTCGAATCGGCGCGTTAAGAGGCCGGTTTTTTCTCCCACCCCAGCTTTGATGGTCGTTCGACCCCCTGATGTGCAAACGGCCGTAACTCGGTCCTTGGTTGTCCGAATGAGGTGATTTCCGTCCCAAATCGCATATATTTCCGAGGTCTACGGAGCCCCCCGGGAGAGCAGGTCGTTTGCCCCCTCGAATCGGCGTTTTAGGGCCCGGCTCCGTGAGCCCATCCACTTTTTATGCTCTTTTCGACCCCCCCGGCAT | 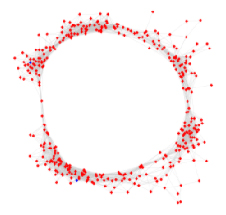 |  |
| 16ch sat4 | *L. decumbens* | 389 | 0.10 | 0.986 | ACATGTAGTAAATAAGACTCCAATTCATAATTCATTTGTTCCATCAAAATATACTCACAAAGAAGTGATAAATTGAGAAAATGTTTTCACGTGAGATGATTTTGATCACGTGGGACAAATTTCACGTTAAGTGGTCCAAGAAAAGTGTCAATGTAAAATGTAATTAGCTTTCCTATTTAGCTCATTATTTGCATTGCCTTGTAAAAAGTTTCATATTTCTGTAAACATAAAAACAGAAATATCCTGCAAAAATTAGCAGACATTGAAATTAGAATACTATACAGAGTTGTTTAAAGTACAACTTGTGAAAGAGATGAAAAGGAAAGATTGTGACTTGAAGAAGTAAGTTGATGAGTTTGAAGAGCTGTTGTGCTTCTCTCTCTCTTTTT | 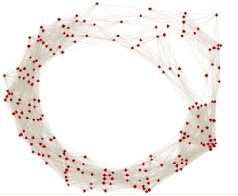 |  |
| 30ch sat1 | *L.usitatissimum L.angustifolium* | 1100 | 1.70 | 0.741 | CAATCCTTCGAAATCGGTGAACACGATTTTGGGCAACTTCCCACCTTCGAATCCAATTACGCTCCGAATGATTCCAAACTCAAGAACATGTCTCTTATCATCATTCAACACCTCTACTCCAAAGATAGTGCGAGCAAAGTTTGCCCGAACTCTCGTTCTTGCGAATCTTGATTCTTGGGTGCGACCGTGACGTTGATGATGATGAAGATGCCGGCGTCGATGCCGTGGTGCAAAATCCGTGCCACTCGTTGGATCGCCAATTCTTCGAGGATGAATTTGTCTTCTTTGATCCAAGGTAGGGACGTCCTTGTATACTTCTAAGAGATCTTCTCTACAAAATGCTTGAGTTGGATCCTTGATTACAAAAAATTGATGGTCTTCTCGTTGAGTGCCTTCGAGGGGTCTCGGGTGTCTAGGACTTTGAAACCTTGTTTAGGAACTGCGGACATAACCTCATCAACTTCCACCATGAACCATATGCTTTCTTACGATGAACATCCACAATGAACCACACATGGTGCACATTAGTACATTACATTTACACTATGCATTGTGCAGCTTCCTATGGTGCACTTCCACCATGCACCATGCACCATGCAGCTTGGAATGGTGCACTTTTACTTTTCACAGTGCCCCTTGGTATGGTGCAATTCTACTAACAACTAAGCAAGAAGGATCTAAAATAAGGCGTCTTGGTCTCCAAGTTTGAATAAAATACTAGATGAAATCGGATAGCATAAGCCTAGGTTTCGTCCTTGATGGTTGGGGCCAATTTGTAGGCTCGTAGGCTGGCCTAAGTGAGGCTTTTGAGTTGCCGATTGGTAGTCTGGCTTGAACCAGGCCTTAGTGACGTCTCATAGGAGTTACTGAACGTGTGCTCTTCATATGGCGCGAGAAAATATGAACCAGGCTCGAAAAGGTGTGAGAGGCCTCGTTCGTTGGCACTGGCATCACCGTAACAAGTCCACCTGTGGGATTCGTTCCCAAACTCCTTTCTCCCTGCTTCCATCGACCTTGCTGGTCTTCAAATAGCCCATTACGCACCTGGTCCAGAGATGGTGCGGGCAAAGTCTTTCGTTTCGACCGGAGTGCGATATGGTG | 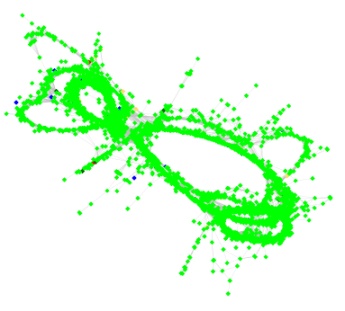 | 0.01% Class_I/LTR/Ty1_copia/Ale:Ty1-RT |
| 30ch sat2 | *L.usitatissimum L.angustifolium* | 311 | 1.70 | 0.975 | AACTTGGTATAGTGCTCTCTCACCTCCTTGTACTTTGCACCTTAGTATGGTGTACTTCCACTATGCACCTTGCACATTAGAATCATGTAGTTCCACCATTCACAATGCACCGTGCCCTATGGTATGGTGCACTTTCATCATGCATTATGCGAATTTGACCCTGGTATGATGCACTTCCACCATGCACAATGGACAGTGGACCTTTGTATAGTGAATTTCCACTATGCACAGTGCACCTTTATATTTTGTAGGTCGACCATGCAATAAGCATTGTGTGCCTTGGTATAGTGCAATTCCACTATGCATAATGT | 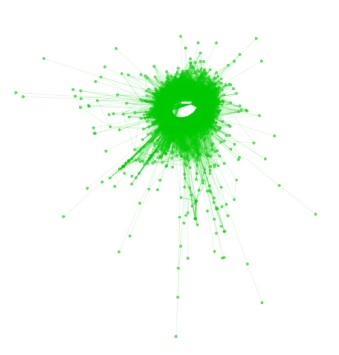 |  |
| 30ch sat3 | *L.usitatissimum L.angustifolium* | 506 | 1.50 | 0.733 | CCTTGTATAATCGTTCACTTTTGTATGCATGGAACCTGTACGCCACCAACACTAGTAGTTGGTACTTTCTTTCTAAAGCTGGAAGACTAGCATCCATATTTAGTAGCTTCATCGTCCATAATGGCTTATGCTCAAGCTTCACTGGTAAATGACAAGCCTTGCCATAAACTAGTGGACAAGGTGAAGTGCCGATCGATAGGAGTCTTGAAGGCAGTTCGAAAGAACCAAAGGGCCTCAATCGAGCTTGATTGACCAATCCTTGAGAGATTGATCGATCGTTTTCTCAAGAATCCGCTTGATCACAAGATTCATCATTTTTTCCTGACCGCTTGAATGTGGTTGGTATGTGATGATCAATCGATGAGTTATCCCAAATAGTTGGCAAACTGGTTAGAAAACACTCTGAAGGTTAGTAACTCGAGCGCATATGACTGAGTGAGTGGCGTCAAAATGTGTCAAAAGGCATTGCATGAACTTTCCCACCGCCTTAGCATCATTACCTTTCT | 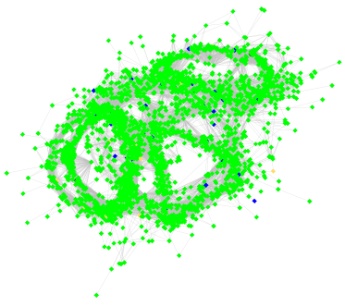 | 34.51% Class_I/LTR/Ty3_gypsy/non-chromovirus/OTA/Athila:Ty3-INT 0.19% Class_I/LTR/Ty3_gypsy/chromovirus/CRM:Ty3-INT 0.09% Class_I/LTR/Ty3_gypsy/chromovirus/Galadriel:Ty3-INT 0.05% Class_I/LTR/Ty3_gypsy/chromovirus/Reina:Ty3-INT |
| 30ch sat4 | *L.usitatissimum L.angustifolium* | 475 | 0.98 | 0.751 | TGTAGCTTCTTAGGGTGCACTTCCAGCATGAACTATGCACCATGCAGCTTCAAATGGTGAGTTTTACTTTGCACAGTGCACCTTCATATTGTGCAATTTGACTGTGCACGTTGCACAATTCACCTTGGTGTGGTGCAATTCCACTATGCACCTTGCACAGTTCACCTTGTTACGATGCACTTCCTCCACGAAATATGAACCATGCAACTTAGTATAATGCACTTCTACTTTGTAGTGTACACCTCGGTATGGTGTACTTCCAACATCCACCTTGCACTTTGCACCTTATTACGGTGGACTTCCACTATCCACAATGCGAGTTAGTATGATGCAGTTGCACTATTCATCATGTACTTTGCACTATGGTATGGTGCACTTCGATCATGCACTATGCTACGTGCATCTTGGTACAATGCACTTCCACAATAAACCATGCATTGTGCACATTAGTACATTACATTTCCACTATGCATTG | 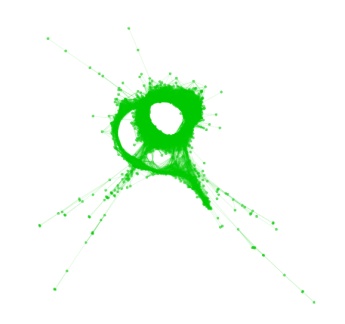 |  |
| 30ch sat5 | *L.usitatissimum L.angustifolium* | 256 | 0.85 | 0.933 | AGTTTCCAGGGAGCATAACCAGAAGTAAATGCGAAGGAAATTTAGTGAAAGACTCATACAGTTAAGGGGAAAACATGGAATAAGTTCCAATTGAAATGCGGAAATAACCAAGTTTAGACAAAACCATACAAGTCTCGTTTTCAGAAAGGAGAAACAAGGGAAAAGCAATAATAAGGGCGGAAATAAGTCAGGAAAAGGGCAGAAATAAGTCGTGATGTGCAGTAGGCCACGAGCATGGTAGACAGGAAACAGAAGG | 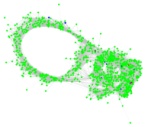 |  |
| 30ch sat6 | *L.usitatissimum L.angustifolium* | 353 | 0.79 | 0.911 | ATCAAAACGTAGTAAAAATTAAGAGAAATGAGTGAAAATGAAGGGAGAAAGTAGTGAAAGACTATTCATGTATATATGAGCGCGAGAAGCAGACGGGTGAGATCATACCACCACTAATTCATCGGATCCCGTCATCATTCCAAAGTTAAATGTACTTGGCTGAGAGTAGTCTAGGATGGGTGACCTCTTGATTTGTCCTCTTGTTGAACGCCTTCTTTTTTCTATTATTGTTTCAATTTTTGCCCTGATGTGTAATGGAAACATTTAACTAGACAACATCCTCTAGTAGAACTTATGTAATGATGTCGGGGATTCGAGAGTGCGGGTTAGTTCGATGCTCCTAGAGAAACGAG | 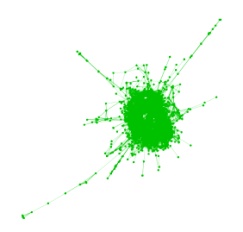 | 11.08% 5S_rDNA/5S_rDNA |
| 30ch sat7 | *L.usitatissimum L.angustifolium* | 105 | 0.76 | 0.992 | TTCATAGGTATTTTTTATAAAAGGTGGACTCGAGCCTTTCTCAGCTGCCTACGTACCCAATACTTTGTGATCAAAGTCCGCGTAGTTCGGGGTTGGAGATTGATTA | 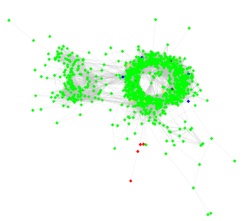 |  |
| 30ch sat8 | *L.usitatissimum L.angustifolium* | 316 | 0.72 | 0.981 | TCATTTCTTAACACGCTAAGCCAATATCGATTAAAATTGATTTTTAGATCCATTTGACTTGTATTTGCAGGGGTTACCATGATGAAACTAGAACTTTTTAGCCATTTCCAACATGCCTATCAAATATTAGCGGATAGGAAACGTTTGAAGTTTTTATGTTAACATACCTCATTTCCATAGGTCACTTGACAAATTTTGAAGTGAAAATAGAAGTTTTCTAAAAAGCCTAGTATTTGACGTGTATGTTGGGAATGGCCAGAAATGGAGGTTTCATCATCTTAATCCCTTCAAATAATAGGCAAATGATGTTAAAAAA | 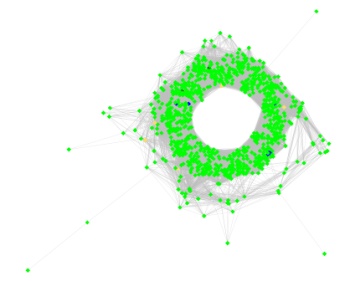 |  |
| 30ch sat9 | *L.usitatissimum L.angustifolium* | 825 | 0.71 | 0.974 | GAAATACATCGATAATGGATATCATCTGATATCAAAACCCCGACCAACTCTCTCTCCACCCTCAGTTCCAGTACCCGTGACTGAAATACAGAGAAACACAAGGGAAAAATGTCTTATGGGGTTAGCGATAAGCACTGATTGGCTATCTATACTCGTGTAGAATTGAGATTGTGCTGAGAATTCAACACGGAAGTCTATCTAATCACTGCATCTAGTGATGTGACCCTATGGTCCGTATAGGAATATACCTAGCATCAGCCACCCGTAACAGTGTGGACAACCTCACCGTTGATAAGACCATGAAACCACCTAAAAAGATTCGTGTGAGCAGCATTTGTAGGCATACTCTACACGTCCTACCAGAATCTGAATATCACTTGAGGGATTGCGCAATCCGGGGGTGGTCGACCAACACATAATCATCGTAAGTACTCTAAATTAGCATATCCTGTCCTAACATGGACGATGAATCACACGCCACAGTTTAAAGGCCAATAGCTTTTGCTCGAAACTTATTAGTTGCATATACAGAGATCACACGATGTATAATCTGAGAGCCCTTGCTCTAATGACATCAACACATTTATACAGTAGATCACACACTACTGTACTATGAAACCTCACCCTAGGAGTCTTGGTCGAACCACTGGTTTAACACAGGAGTCCACCTCAGGGTGCAAGTTGAACCAACCTCGACTCAAGACCTTTGATAAATCCAATAAACAAGCATTAAAATCTGAACTGGAATACTTTAAATAAATTGACCAATAATGATCTTGGAATCCCTAATGAAAATAAAACAAAAGCAGATACAACTCCAGTTCA | 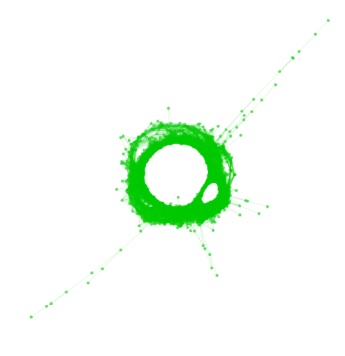 |  |
| 30ch sat10 | *L.usitatissimum L.angustifolium* | 442 | 0.63 | 0.981 | AACCTCAAACCTAGTGTTTTTGTAGGATTATGTGCACTCCTTGAGACTCTTTCGTGTGAAGATCCAGCTAGCTCAAGAGTGTAGAAACCAAAATACGATAGAGCATAACGAAAAACACGAAGTACTTGAATAATGATTATTTTTTGATCCTATTGTTTGAGATACAAGACGCAGACATCAACTTTAATACCATGAAGACGACAAACAAAATAGATGGGGACGATAATCGAAGTTTTTGGTTCAAACAATTGGACTGGTCTATATAGAGTCTTAGTATTTGGAATCCATAGGTTTGAGAAATCATGAAAATCCATGTTTACACAACGCCTTCTCCACCTCAAAACTGCTCAGAAATTCAATGTTCAACTCCTCAAAATAAATTGTGAATTGATTAGATGATAGCAAGCTTGTTTAAGAATGTACTCTCCATCTCTCTCGCTCT | 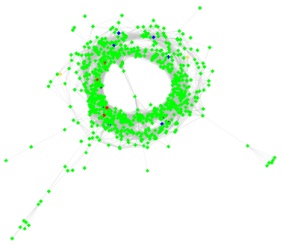 |  |
| 30ch sat11 | *L.usitatissimum L.angustifolium* | 795 | 0.55 | 0.923 | ACAACTTACTTTTAGGTTATATACTTTTTTGAAGATATGAATTCCTATATGAAATATAGTTTTACTTACGCATATAATTATAGATATATGAAGTCTAGATTTTAGGATTTTTAACTACTTTTTAATTCTATATACGACCAGTATTATCCGGTTTGATTATTAATTCATCCGTACGTTATTTTGCTATTAATTGTCATATATATTTTTTCTTTATTATTACAACTAGTTTAAACGGTAACAAAACCACGGTTGAACCATCAAATTTCATACCACTAAACTTTCCGATATTGTGGCCGGTACGGTTATTAAAACATTGCTTTTAAGAGGTATGTACTCCATTTCAAAAGTGGAGAATCCCAAATTATTCACTTTTCATAAATATAGCCAACTACGGTGAGACGTTAACCGTAAAAGGGCATGCAGCTGTATGCATGACTATCTCATACGCCATGTGTGGGAACATTATTAATTACCGTGCGTCCATACTTCTTATCGGTCAACCTTATCATTGTGTACTTTACCACCAAAATATATGAAGTTCATATCATTTTAGTTCGATTTACTTGACGAATCTAAAAGAACAAATCTAGACATCAAATTAAATATACAATGCAAACCTATCCTCAATTAATATGTTAGCCTCCATATTGTGATTATATTTTTCCAGCTACATAATTAGATATAAAAATTAAATAGTATTATCTCCACTAACATCAGTCTAATTACACAAATAATTTTATTTATTTTTAACAAATACATTCTTTTTTCAATTAAAAAATAGAATTGTATCATATC | 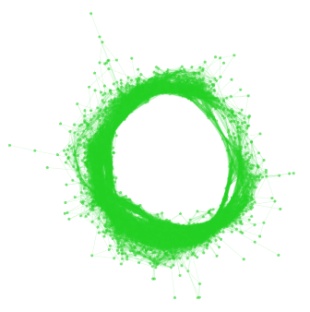 |  |
| 30ch sat12 | *L.usitatissimum L.angustifolium* | 458 | 0.52 | 0.979 | TGTGGAGGGTGATTTGACAACTCTATTAGTAATTGGTAGGAATGTAAGAGTGATTGCTAATTAACAAATTCATTAGCAAAGCCACATAGAATATGAACCAATTTCCTATGAGAAAACTTTATATTAACCATGATACATAAGCATATTGGAACGTTTTTCTAAACATAAGAAATTGCTAAATATTTTAGAGATGTAAGTGATTAGAAATTGTTTAAAGAATGAATTGCTACAATTAGTGATGTGAAAGTTAAAAATTACTAAATAATTTCTAAGATATACATGATCTTAAAGTCCATTTGAAACAAAATTAAATTCTCCATATTCTGGCATAGTTTCTTATGTAAAAAACTTGTGATTGCTTGTAACCAATTTTGATTTTTGTTGTTGTGTACTTATAGCATAAATAACAATCACGTTCAGCTCTTGTACATCCTTAGTGCTTTATAATTGTTAAGTGA | 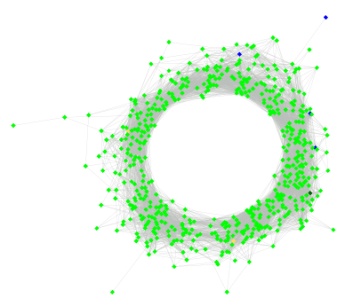 |  |
| 30ch sat13 | *L.usitatissimum L.angustifolium* | 259 | 0.52 | 0.986 | AAAACCGATTAATATTATACGTTTCCCATTCTACCCCTTATACTCTCTCTCCTACCCATCACGTGAAATCCAACCAATTGAAGTCCAACGATAGAGAACGTATCCGCGCCAGCGGATAGAACCGTATATCCGGAGAATAGAAAAATTGAACTGAACAGACATGATGATGATCAGCCGTACCATGATTGTCGATAACATAATACACTTCATTTTTCTTTTATTAAAATTGAAAATAATGGCTCCGAGAGAATTTTCCCGC | 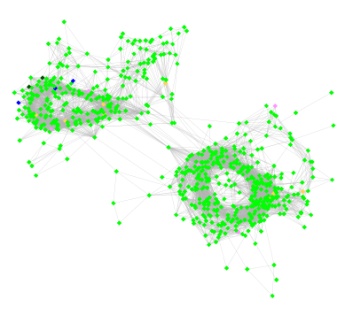 |  |
| 30ch sat14 | *L.usitatissimum L.angustifolium* | 790 | 0.37 | 0.981 | CTTAGACTATCCACACTGCACCTTGGAACGGGGAACTTCCACCATCCACCTTGCACTTTACTTGTTCGTACGGTGTACTTCCACCATGAACCGTACACCTTGATACAATGTACATCCGCGATGACCCATGCATGGTGCACATTAGTGCATTACATTTCCACTATGCATTCTGCAAGTTGGTATGTTGCACTTCCACCATGGACTACGCACCATGCAGCTTGGAACGGTGCACTTTTACTTTTCATGGTACACCATGGTACGGTGCAATTCGACTATGCACCTTGCATAATGCACCATGGTATGGTGTAATTCCATTATGCACCATGCACAATTCACCTTGGTATTGTGCAAATCAGCTATACACCTTGCACAATTCACCTTGTTATGATGCACTTCCACCGAGCACTATGGCACGTGCTCCTTGATATAGTGCACTTCGACTATGTAGTATAAACCTTGGTATCGCGCACTTCGACTATCTACCTGGAACTTTGCACCTTAGTATATTGGACTTCCACTATGCACCATGTATATTAGTATGATGCAGTTCCACTATTCACCATGCATTGTGCACTATAGTATGGTGCACTTCAACCATGCACTATGCATTTTGCAACTTGGTACGACGCACTTCATAACACACTATGCATCGTGCAGCTTGGTACAGTGAATTTCCACTATGTACCTTGCACTTTGCACCTTGGTACAGTGCACTGTCATTATAAAAACTGCAACTTTGTATTTTTCACATCCACCAAGCACTATGCTCTTTGAACCCTGGTATAGTTCA | 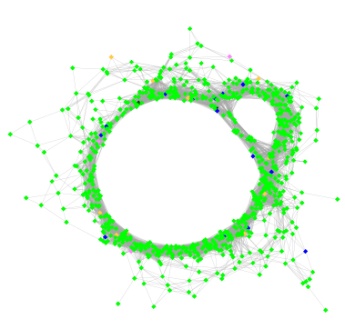 |  |
| 30ch sat15 | *L.usitatissimum L.angustifolium* | 392 | 0.36 | 0.903 | GCCTTGTTATGGTGCACTTCCACCTTGCACTATGTACTAAGCAAATCGGACTGGAGCACTTCCACTATGGACGCAACACTTTCGTATGGTACACTTCAACCGTCCATCTCGCACTTTTCACCTTATTACGGTTTACTACAACTATGCACTGTTGAAATTAGTATGCCACGGTTCCACAATTCACTATCTCCCATGCACTATGGTACGGTGCACTTCCATCATGGACTGTGCACCATGCAGCTTGGTACGGTGCCATTCTGCCATGCACCATGCAACTTGAACCTTGGTACAATGCACTCCCACTAAACACATTGCACCATGGTTTTTTTCACTTCCACCATGCACTATGCACCGTGCAGCTTGGTATAGTGCACTTGCACTATGTAGTGTGG | 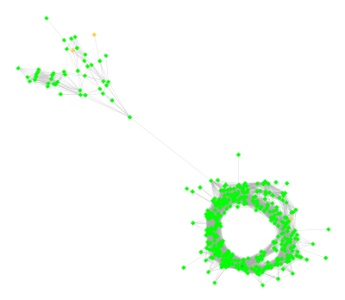 |  |
| 30ch sat16 | *L.usitatissimum L.angustifolium* | 444 | 0.35 | 0.895 | ATGGGGACGTTAACAGAAGTTTTCGGTACGGACAATGGAATGGGCCTTTATAGAGTCTGAGTATTTGGAATCCATAGGTTTGGAACCATCATGAAATTACATGGTTGAACACCGCCTTCTCCAACTTAAAACTTCTCAAAATATTTTATGGTCAACTCCTCAAAACCAATTATGAATTGAGAAGAAGATAGCTATCTTATTTCAGAATGTATTCTCTTTCTCTCTCGATCTAACGTCGAACCTAGTATTTTTGGATGGTTTTGTGCACTCCCCGTTACTCTTTCGTGTGAAGTTCCAACTAGCTCCAAAGTGTCGAAATAAAAATACGACAGAGCATGATGAAAAACAAGAAGAACTTGAGGGATACCGATTTTTTGGTACTAGTGTGAGAGATACAAGACGCCAACGTCATCTCTAATACCAAGAAGACGACGACCAGAAGAG | 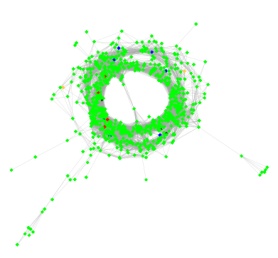 |  |
| 30ch sat17 | *L.usitatissimum L.angustifolium* | 645 | 0.34 | 0.974 | GAGTTTATCACTATTTACCTTTTGAAGCGTGAGTACTAGCTTCACGACTAATACTTCCTGAATGCTTTAGATTGGTCTCGAGAGCTTCACATGTAGCTCATCTACTTATTCCATGCTAGCTGTGTTTTGGGTCTTCTTCCTCAAAGCTTGCTAAATCTCTTAATTGAAGAACGAATCTTGAGAGCATATGGTGGCTAGTGTTTATCTTTGTGAAGGCCGCTCAAGGCTTCGAATAAACTCTTATTAATACCCATATCATTATAGGGTTTCTACTAAACTCTAACCCTATTGGGGCACTAACTTACTTACCCTTACCAATATTAGATTAGGATATTGATTTGTTCCATACTATATGTGTGTAACCTTATAGGACCTCAGATTATCCGTGGCCCTAACCACTAATTAATAGTGGCCTCTAGAAAGACATGTTAACTCCTAACCGTCCTGAATTAATTTTGATAACTCGGGTCTATCACTTCACTAGTACTCATGCGGACTTTGACGGAGGGACTTAAATCCTTCGGTAAACTAGGCTCTGATATTAGTGTCATAAATCGTTTAAATGAAAATAACAATTTTGCAACGGAATTACAAGAACATGCAACATTGTATTTCAACTCGTATGGAAATAAATCAACAAACAAA | 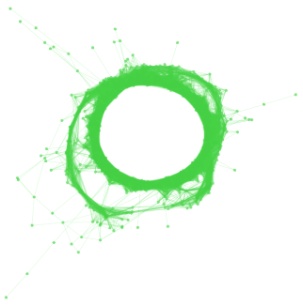 |  |
| 30ch sat18 | *L.usitatissimum L.angustifolium* | 556 | 0.22 | 0.992 | AAAAATTAGGAAATGGTGCTCAGCAGATTTTGGTAACTAGTTAAAGAAAAAATCAAAAACTAAATCATGATTTTTGAATTTCACGAATAGGGCCGAGAGCAAATACGAGGAACTAGGTCGAGAAGAAATGGTAGGAATTGAGTCATGACAAAATTTTGGGAACTCCAAATTTTAGGAACTTGGTCGGGACTAAAATTAAGACCCTACCTCTATGTAAAGATAGAGATAGAATTGAAAAATGCCAAAGGATCGAGATACCACCAACTACACATGTATTTGGGTCGATTGGGTCGGATATGACCATTACCAAGTAACTAAACCATAGCTCTCAGTTTACAAAACATAGCTCTCAATTGACAAAATATCCTACACATACCCAACCATCAAATATAAAACGCAAGGATTAATTGAAGCAATAAGTTTCTTTTTTATAATATTATCCAAAAATCTTTAGGATGGTAGAATTTACCAAAATGTATTGTTTTTACTTTCTCTAATGTATAACACTCTCTTATGATTAGTATTTTATGCCTAAATAAAATTATTTATTCATATA | 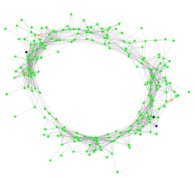 |  |
| 30ch sat19 | *L.usitatissimum L.angustifolium* | 114 | 0.22 | 0.781 | ATGCGTCTTTTATTATGTCGTTGTTACGTCCACCTTACGAGTAGTTGAATTCATCATCGTTATGTCCAGTTTATAACTCTTTTATGTCGGTGATATACACTTTACGAGTATGTT | 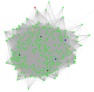 |  |
| 30ch sat20 | *L.usitatissimum L.angustifolium* | 432 | 0.20 | 0.981 | GAATAAGGTTATCTTCGAACATCTGCAACTGTCGATCGACTTCCTGGCAAGGCAATTCACGTTTCCCTCCCTCACATATACCTCACGTTTTCATCGGCCATACATACATCTAAGTTTCTAATAACTTACGTCAAAGTTGCAATACCTACGTTTAAGTTGCTAATAACTACGTGTAACTAGACAATAACTACCTCTAAGCTCTCAGAAGATAGAGGTCGATACAATGTGTATAGTTTGTGGCATTGAAGAAGAAACACAACATCACCTATTCTTCTCTTGTCTAGTGGCGATTCAACTGACCATCAAGTGTGGTTGTCGTCATTTCATTTCGGGGACTGAGCATCTGATTGCCTTCGGGTGGCGTTGCCATAATTTAGATCTGGCATCATCCATCAAATTGATTTACTACTGGTGGAGGTTGTGGAAGTCTCG | 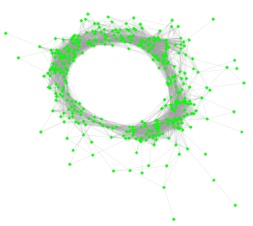 |  |
| 30ch sat21 | *L.usitatissimum L.angustifolium* | 510 | 0.12 | 975 | ATTCTTATTCCACCTAGTAATGTTCTAAGTGAGATTCGTATAGCTAATGTTCTTAAATTCATAAAGTAAATACCGTAACGTAATTCGTAACATAATATAAAATAACAAGATTAAGGATTTACCCCTTTAAACCATCCACTTGATAAAAAAATGGTGAGGGTCCAATCCTAGGGGTGAAGGTTTAAGCTGAAAGACTTGATCGTCATTAACAGCCTAAAGACGAGGATTTCAGATATTAGACGTAATTCCAACATGGGTTGAAATAGTCTGGAATGACGACTTAGCAACTTTCGAGGAATGAGTTCACAAATAAAGTGATGTGGCTGAAAGGATTGATCCGAAAGAACTTGTGTTCCAAGGATCTAACGGATGTAAGAAGTATGTTATTATAATGTCTTAAAAATTCTAGTTATCAGAGAGAAAGGAGTTCATAAATTTAATTACTAACAATGGAAATTCATGAGCTAATACTTAATTGAATTCTAATTATAACTTACTAGTGAGGTTCAT | 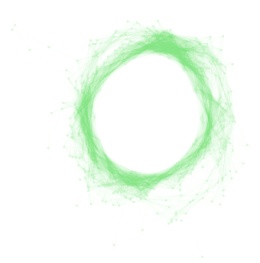 |  |
| 30ch sat22 | *L.usitatissimum L.angustifolium* | 410 | 0.097 | 0.994 | AAGTTGGAGGAATATCCATTAAGACAATGGGAGTATTACAATTAGCTATACAATGCGCCATAATATGGGAAACTTCATTCACTTTACGGTAACCATGATTCCAAACACAAGTGTCAAACTTAGGGAACGACTCACGAATGCTTTTACAGATGATCCCCAACTCCGTAATATTATCATCCACATTATGTAAGCCCTACCACACTATCAAGTTATCACTTTTCACAAGAACCTTACTTAATCGATGGCTGAAAGCAAGTTACAAACTGAACTCCACCACGACTGCTTCTGCCATATCCGACATCCAACTCCCTTCGGTTCTTAAACTTTTATGATTTTATAAAATGAAACTTGTAAGATATTATTAATCTAAATTAGGTCTTAAATCGTTCAGCTTATGATGGTCAATCAGA | 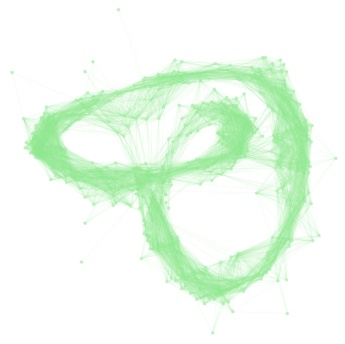 |  |
| 30ch sat23 | *L.usitatissimum L.angustifolium* | 433 | 0.082 | 0.802 | CGTTCCGCACATTTTCTGCTTTCATCCGCCCTACACCCTCTAAACTCTGGTCTTCAACATACTCCAAATTGTATTGTTGTACACGTTTAAACTGCTTCTGGAATATTTTGAACACCTTGGGTGTGTACTCCTTAGATGCTTTGGTCACTAAATTACTGTACTCAAAATTATTTATAGGTGTTGTGTTCGTAGGATGGAAGTAAGTTTCCCGCACTGCATGTCTTCTGCTTTCAACCATCCTACGTGTAGAATCACAACATAAACACATTAGAATAAAAAAATAATTATCACAAAACGATAACATATTTATTATTAAACATCCTGGTTCTTGAGCGAACATATTGGGTGTGTACTCCTTGGATGCTTCGGTCACTAAATTATTGTACTCAAAATAATTAGAGGGTTTGTGTTCCTAGAATGGAAGTCAGCTTCC | 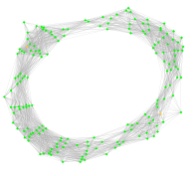 |  |
| 30ch sat24 | *L.usitatissimum L.angustifolium* | 803 | 0.036 | 0.981 | GGTTTGACAATTTAGTCCATAACTGTTAATTGCCATTGTCGCGGCCTGTAACTTTCAGGTTTTGACGGAATGCATCCAAATAATATGCTAAGTAGTACAATCTAGTACAAACCCGGTTAATTATTTGTTATTGAATATTAGACAATAGAACTGATACTTGATAAAGTTTTAGTTGTAATTGATATCGTTAGTATGAAATTTATTAGATATTAGGAACAAATAAATGTTGATGATAGTTGGATAATATAATAAGATTTGAATTATGAATTAGTCATTATTGATAGATAAGAGGTGGATTTTGTAGTCTACAAATTCGACATCATTAATATTCCACTTTTTCTAGGATAACTAAATCATAAAATCGAATTATAATATGATTGAGATAAAAATAATTAGTAGGCAATAATTATCGTTAAGTGAACTTGTTTGTCACACTTCACAACCGACGGTGACGTGGCTTTTTCCATCTATGTTTTTTAAGACGACGGATCCGAAGATACCAATCATCGAGACTGGTTTAGGTCCTCAAGTTCACATTATTAACTTGTACGAAGGTTAGGAAGCGATTCTATTTATGTCTTTTTGCTTTGGTTGAGTTATTATGCGTATAGGCTTAGGCAGAATCTGGGATTTTGGCGCCGTATTCTTGGTTCTTGATGGTTAAACGACGGCGGTCAACATGGTGAAAGTATTTGTAGCAGTCCCTAGATGTTTCAGTTATTACAATTTGGTCACTGGACTTCCAGCAATTCTTTTTAGTACCTATAGTCCTAGAAATTATGTTACAAATAGAGTAGTTCTAG | 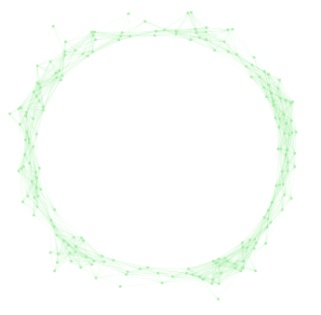 |  |
| 30ch sat25 | *L.usitatissimum L.angustifolium* | 326 | 0.028 | 0.986 | ATATATATTATGCATCATATCCAAAAATTGGAAGGTTTTGAGTCTGACAAAAATTTTGAATTCCTCTTCGGAACTGTTTAATGTAGCTAAACTCACCCGCCCGATGAATTTCGACCAAACTCCAAACCTTCCAATTTTTGAATATGACGCATAATATGTGGTTAAATGATGATTGGGATAGGTTTCGGTTATTAATCTTCGAAAATGATGGCTAAAACGGATGAAATTAGTTACATTGACTAGTTCCGAATAGGAGCACCGGAATTTCGACCGACTCCAAACCATTCAATTTTTTTAATAGGAAACCTATCTCAACCACCATTAAA | 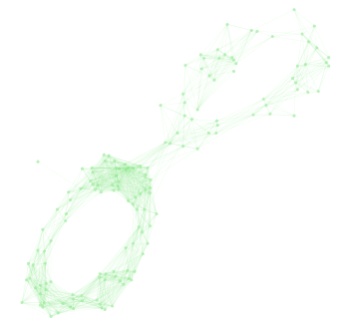 |  |
| 30ch sat26 | *L.usitatissimum L.angustifolium* | 266 | 0.013 | 0.986 | GTTCCCATCAAAACTAAGAAGGATCTTCGGCAAGGTGATCCTTTGCCACCCTTCTTCTTTATCATTATTTTGGAGGCTCTCTCTTGCATGCTTGATCGTAGTGGGGAATGGGGTGCATTTTCTTTCCTTCCCTAATGTCAAGAGATGCCATTGACCCATTTGGCTTTTGCCGATGAATCCCTTAGATGGGCTCTCAAAGTGGACCTAATGAAGGCGTTTCACTCGAACAATTGGGATTTTCTCTTCAACATCATGAAGGTCATGAA | 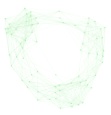 | 30.43% Class_I/LINE:LINE-RT |
| 30ch sat27 | *L.usitatissimum L.angustifolium* | 583 | 0.8 | 0.531 | CAATTGGATGCGAAGGTGCGAAGTTGCTCCAAATTGGGTTAATTGGTTTCGATGGATCGCACCCAATCGCACTCCGACCCAAATGAGAAGACTTTACCCGCACAATCCCCGGATGTGGTACTTAATGGGGTATTTGAAGACCGGCAAGGTCGATGGAAGCAGGGAGAAACGAGCTTGGGAACAAAGCCCACATGTGGACTCGTTCCGGTGATGCCGGTGCCACCGAAACAAGGACTCTCATACACTTTCGAGCCTGGTTCATTCTAGATTTAATTTTGTGGAATAAAGGTTCCAACCCAAGCATTTTCGAGAGGCTATCTCTTAGAAGGATACAAGGACGTCCCTAGCTTGGATCAATGAAGGTAAATTCATCCTTGGAGAATTGGCGATCCAATGAGTGGCACAGATGCTGCACCACGGTATCGAGTCCGACCTCGGTACCAGCATAAACATCACAGACGCATCCAAGAATCATGATTTCCAAGATCGAGAGTTCGGGCCAACTTTGCCCACACTATCTTTGGACCAGAGTTGTGGAATGATGATTAGAGACATATATTTGAGTTTGGAACCCTTCGGAGCA | 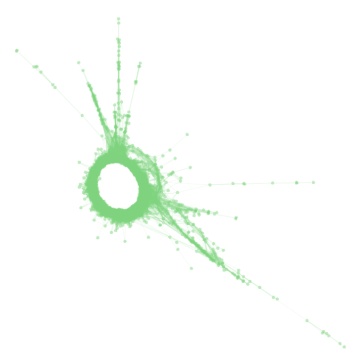 |  |
| 30ch sat28 | *L.usitatissimum L.angustifolium* | 385 | 0.16 | 0.644 | ATGATGTTGCACTGTGCATAGTCAAAATCCATTATTGTAGTGTACATTGTGCATGTTGAAAGAGAATCGCACCAAGACGCAAAGTGCAAAGTTGATGGAAGAAGCGCACCAAACCACGGTACACTATGCATGGTGGATATGCTCTATACCAAGGTTCACGGGTTATAGTGAATGGTGAAAGTGGAAAATAGCAAGGTGCACTATGTATGTGCGAAGTGCACTGTACCAAGATGCAAGGTGCATGGTGCGTAGTGGAAGTGCATCGTACCAAGGTAAAATGTGACTGGTGCATGGTGTAATCGCACCATACCAAGGTTCACCGTGCATCGTGGAAGTGCATCATTCCAAGCTGCATAGTGCATAGTGCATGGTGGAAGTGCACCAC | 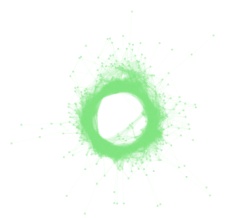 |  |
